# Defects in autophagy lead to selective in vivo changes in turnover of cytosolic and organelle proteins in Arabidopsis

**DOI:** 10.1093/plcell/koac185

**Published:** 2022-06-29

**Authors:** Lei Li, Chun Pong Lee, Xinxin Ding, Yu Qin, Akila Wijerathna-Yapa, Martyna Broda, Marisa S Otegui, A Harvey Millar

**Affiliations:** Frontiers Science Center for Cell Responses, Department of Plant Biology and Ecology, College of Life Sciences, Nankai University, Tianjin 300071, China; ARC Centre of Excellence in Plant Energy Biology, School of Molecular Science, The University of Western Australia, Crawley, WA 6009, Australia; ARC Centre of Excellence in Plant Energy Biology, School of Molecular Science, The University of Western Australia, Crawley, WA 6009, Australia; Department of Botany and Center for Quantitative Cell Imaging, University of Wisconsin-Madison, Madison, Wisconsin 53706, USA; Frontiers Science Center for Cell Responses, Department of Plant Biology and Ecology, College of Life Sciences, Nankai University, Tianjin 300071, China; ARC Centre of Excellence in Plant Energy Biology, School of Molecular Science, The University of Western Australia, Crawley, WA 6009, Australia; ARC Centre of Excellence in Plant Energy Biology, School of Molecular Science, The University of Western Australia, Crawley, WA 6009, Australia; Department of Botany and Center for Quantitative Cell Imaging, University of Wisconsin-Madison, Madison, Wisconsin 53706, USA; ARC Centre of Excellence in Plant Energy Biology, School of Molecular Science, The University of Western Australia, Crawley, WA 6009, Australia

## Abstract

Identification of autophagic protein cargo in plants in *autophagy-related genes* (*ATG*) mutants is complicated by changes in protein synthesis and protein degradation. To detect autophagic cargo, we measured protein degradation rate in shoots and roots of Arabidopsis (*Arabidopsis thaliana*) *atg5* and *atg11* mutants. These data show that less than a quarter of proteins changing in abundance are probable cargo and revealed roles of ATG11 and ATG5 in degradation of specific glycolytic enzymes and of other cytosol, chloroplast, and ER-resident proteins, and a specialized role for ATG11 in degradation of proteins from mitochondria and chloroplasts. Protein localization in transformed protoplasts and degradation assays in the presence of inhibitors confirm a role for autophagy in degrading glycolytic enzymes. Autophagy induction by phosphate (Pi) limitation changed metabolic profiles and the protein synthesis and degradation rates of *atg5* and *atg11* plants. A general decrease in the abundance of amino acids and increase in secondary metabolites in autophagy mutants was consistent with altered catabolism and changes in energy conversion caused by reduced degradation rate of specific proteins. Combining measures of changes in protein abundance and degradation rates, we also identify ATG11 and ATG5-associated protein cargo of low Pi-induced autophagy in chloroplasts and ER-resident proteins involved in secondary metabolism.

## Teaser

Protein cargo of autophagy in plants can be discovered by identifying proteins that increase in abundance and decrease in degradation rate in mutants deficient in autophagy machinery.

IN A NUTSHELL **Background:** Autophagy helps maintain the health of cells by degrading cellular components including both functional and structural proteins that are not wanted. If autophagy is inhibited in a plant cell then accumulation of autophagy cargo might be expected. However, proteins can accumulate for a variety of reasons as plants respond to the loss of autophagy; they could be cargo or they could be stress responses or they could be components of alternative pathways for protein degradation. To find which proteins are autophagy cargo, a combined analysis is needed to determine whether a protein is a direct autophagy cargo or an indirect cellular response to autophagy disruption.

**Question:** Are proteins that accumulate in deficient autophagy mutant genuine autophagy cargo? We wanted to find out a better way to define autophagy protein cargo that did not just measure their abundance and/or presume that transcript abundance indicates protein synthesis rate.

**Findings:** We find over a hundred proteins that satisfy a new definition of autophagy cargo by both turning over more slowly and accumulating in the Arabidopsis autophagy mutants *atg5* and *atg11*. About half of these proteins are unexpected autophagy cargos including glycolytic enzymes and many other cytosolic proteins. To provide confidence that our way to find new autophagy protein cargos really works, we chose the glycolytic enzyme fructose bisphosphate aldolase 8 (FBA8) to assess by traditional autophagy protein cargo assays. Protoplast assays showed FBA8 tagged with fluorescence can be engulfed by autophagic bodies and delivered to vacuoles for degradation in wild-type but not autophagy mutants. Stopping autophagy by chemical inhibition also stopped FBA8 degradation, supporting our method to find new autophagy targets.

**Next steps:** While we find a range of new autophagy protein targets, the next step will be to establish how they are recognized by autophagy for delivery to vacuole for proteolysis.

## Introduction

Autophagy enables cellular sugar, lipid, and protein recycling and maintenance through the trafficking of cellular material into the hydrolytic environment of the vacuolar lumen. Autophagic degradation involves general and selective processes and is controlled through both autophagy-related genes (ATGs) and a range of receptor recognition mechanisms (An and Harper, 2018; Marshall and Vierstra, 2018). Large protein complexes like ribosomes, proteasomes, and protein aggregates are recognized through receptors–adaptor interaction and engulfed by autophagosomes for delivery to vacuoles (Marshall et al., 2015; Floyd et al., 2016; Jung et al., 2020). Autophagic degradation is also involved in clearance of chloroplasts, mitochondria, peroxisomes, and endoplasmic reticulum (ER) during developmental transitions or stress responses (Liu et al., 2012b; Farmer et al., 2013; Li et al., 2014; Khaminets et al., 2015; Izumi et al., 2017; Zhang et al., 2020).

ATG proteins participate in autophagosome induction, membrane delivery, vesicle nucleation, cargo recognition, and phagophore expansion and closure (Marshall and Vierstra, 2018). While some ATGs are encoded by single or duplicated genes in plants, there are notable exceptions like ATG8 and ATG18 which are encoded in multi-gene families (Thompson et al., 2005; Xiong et al., 2005; Yoshimoto et al., 2009a). In the phagophore formation, ATG8 and ATG12 are each typically activated by ATG7 and transferred separately to ATG3 and ATG10, respectively. Subsequently, ATG8 is covalently attached to phosphatidylethanolamine (PE) and ATG12 is attached to ATG5, forming an E3 ligase complex. The ATG5–ATG12 conjugate mainly helps drive ATG8 lipidation and contributes to phagophore expansion and maturation. *ATG5* mutants in Arabidopsis (*Arabidopsis thaliana*) fail to form autophagosomes, show a general disruption in subsequent autophagy-related processes, and senesce under nitrogen- and carbon-limiting conditions (Thompson et al., 2005; Yoshimoto et al., 2009b). ATG11 is an accessory protein that aids the scaffolding of the ATG1 kinase regulatory complex to the expanding phagophore. ATG11 is reported to promote vesicle delivery to vacuoles by stabilizing the ATG1/13 complex, but does not appear to influence ATG12–ATG5 or ATG8–PE conjugates. Arabidopsis mutants deficient in *ATG11* also senesce rapidly under nitrogen- and carbon-limiting conditions and fail to degrade mitochondrial proteins during dark-induced senescence (Li et al., 2014; Li and Vierstra, 2014).

The apparent accumulation of specific sets of proteins in *atg* mutant plants (Avin-Wittenberg et al., 2015; McLoughlin et al., 2018; Have et al., 2019; McLoughlin et al., 2020) may be caused directly by a failure in autophagy-dependent protein degradation, or indirectly through an increase in protein synthesis rate due to their enhanced transcription or translation. A failure in protein degradation could also be accompanied by lower levels of protein synthesis through either feedback attenuation of transcription or translational control. Thus, using steady-state protein abundance as sole criterion to identify autophagic protein targets is prone to errors (Wijerathna-Yapa et al., 2021). A couple of multi-omics studies surveying the effect of autophagic recycling on proteome remodeling attempted to use comparisons of mRNA and protein abundance in maize (*Zea mays*) *atg12* genotypes to resolve this issue (McLoughlin et al., 2018, 2020). These studies found that more than half of the proteins that accumulated in *atg12* plants did not have consistent changes in the abundance of their mRNA. Using a similar approach, a lack of correlation in protein–transcript changes was also observed in *atg5* plants (Have et al., 2019). In addition, differential regulation of translational rates implies that the same amount of mRNA will not always result in the same level of translation, especially in autophagy mutants in which ribosome accumulation has been reported (Gretzmeier et al., 2017; McLoughlin et al., 2018). It, therefore, remains an open question as to which proteins that accumulate in *atg* mutants are actual autophagy cargo and which are autophagy-related changes in protein synthesis rate.

Autophagy deficiency also leads to changes in the abundance of metabolic intermediates in plants. Metabolic profiling shows that *ATG*-deficient mutants respond differently to prolonged darkness or nutrient limitation by undergoing extensive rearrangement in primary and secondary metabolism (Masclaux-Daubresse et al., 2014; Avin-Wittenberg et al., 2015; Barros et al., 2017; McLoughlin et al., 2018; Have et al., 2019; McLoughlin et al., 2020; Barros et al., 2021). These reports show that the changes in metabolic profiles and protein abundance in *atg* lines following nutrient limitation is complex, depending deeply on environmental, developmental, and tissue/organ context.

Our previous use of stable isotope progressive labeling to measure protein turnover rates in barley (*Hordeum vulgare*) and Arabidopsis revealed that organelles and intra-organellar components are degraded at different rates (Nelson et al., 2014; Li et al., 2017). These rates resulted from the combined action of specific proteases in different subcellular compartments, the proteasome, and autophagy-dependent and autophagy-independent vacuolar degradation. In this study, we combined a quantitative analysis of changes in protein abundance with a stable-isotope progressive labeling strategy to measure protein degradation rates in *atg5* and *atg11* lines of Arabidopsis. Using these data, we quantified the contribution of autophagic degradation to the clearance of different organelles under control and phosphate (Pi)-limiting conditions. Changes in protein abundance and turnover rate provide clues to understand broad changes in metabolite levels in autophagy mutants, links between cellular trafficking and autophagic flux, and to identity a range of autophagy target proteins in shoot and root tissues.

## Results

### Arabidopsis *atg5* and *atg11* mutants do not show accelerated senescence in hydroponics at early stages of leaf production

We chose a developmental stage prior to leaf senescence in *atg5* and *atg11* (Yoshimoto et al., 2009b; Li et al., 2014) to avoid senescence-associated protein abundance and degradation rate changes from dominating our analysis. By 21 days after germination, wild-type (*Wt*) and *atg* mutant plants showed no visible signs of senescence and had developed 10 rosette leaves, resembling growth stage 1.10 plants as reported previously (Boyes et al., 2001; Supplemental Figure S1A). Consistent with the visible appearance of plants, the quantum efficiency of photosystem II (PSII; Fv/Fm) was the same in *Wt*, *atg5*, and *atg11* leaves, and no evidence of early senescence hot spots were observed in pulse-amplitude-modulation (PAM) fluorometry images (Supplemental Figure S1, B and C).

### Deficient autophagic flux leads to broad changes in the abundance of proteins in Arabidopsis roots and shoots

Changes in relative protein abundance between different genotypes and their biological replicates were measured using a ^15^N reference sample as a control. Total root or shoot proteins extracted from *Wt*, *atg5*, and *atg11* grown in ^14^N media were mixed with equal amounts of reference samples of ^15^N fully labeled *Wt* shoot or root protein (Li et al., 2017). The combined samples were digested by trypsin and the resulting peptides fractionated and analyzed by mass spectrometry (MS). In total, 25,771 nonredundant peptides from root tissues and 18,939 peptides from shoot samples could be quantified using ratios of ^14^N sample peptides to ^15^N reference peptides. These peptides mapped to 1,265 nonredundant proteins in roots and 777 in shoots, which represent high abundant proteins that could be quantitatively compared between *Wt* and *atg* lines (Supplemental Data Set 1; Supplemental Figure S2). We performed pairwise comparisons between *Wt*, *atg5*, and *atg11* using protein sets that were quantified in all three biological replicates. Volcano plots showed that both autophagy mutants exhibited symmetric distributions for sets of proteins increasing or decreasing in abundance. Fold changes (FCs) in protein abundance in *atg11*/*Wt* show a relatively narrow range of FC (two-fold FC in root and four-fold FC in shoot) compared with a wide range of FC in *atg5*/*Wt* protein abundance (four-fold FC in root and eight-fold FC in shoot) (Supplemental Figure S3). A label-free quantification (LFQ) method yielded a higher number of proteins that could be quantified and shown to change in abundance (Supplemental Figure S4), but the overlap correlated very strongly with the ^15^N reference peptide quantification analysis (Supplemental Figure S5). To enable more direct comparison to the later progressive labeling experiments, we used the ^15^N reference peptide quantification as our primary source for protein abundance analysis and refer to LFQ data when needed in supplementary figures.

**Figure 1:**
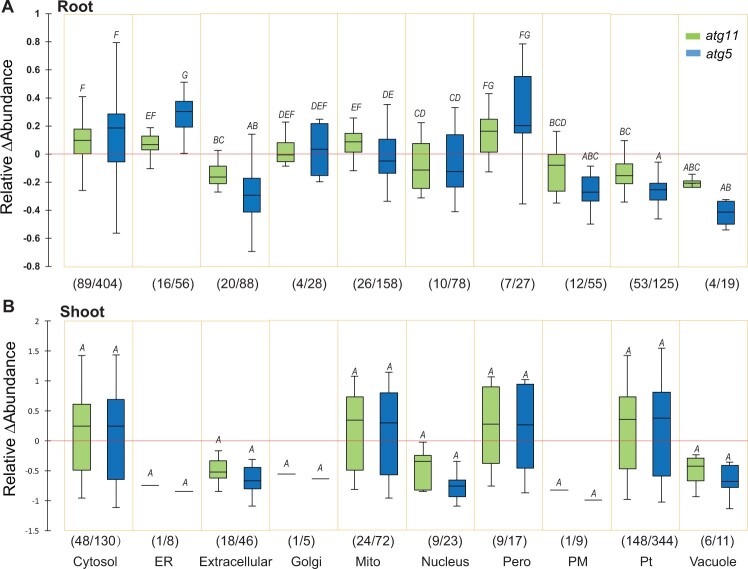
Changes in abundance of proteins in *atg5* and *atg11* that are resident in different subcellular locations. Box plots of relative changes in abundance of 241 root (A) and 265 shoot (B) proteins from *atg5* and *atg11* which significantly changed in abundance in comparison with *Wt* (*P* < 0.05). Box plots indicate the median (center lines), interquartile range (borders of boxes) and minimum and maximum values (whiskers). These were from the larger set of 1,114 root and 698 shoot proteins that were quantified in all genotypes. ΔAbundance changes of specific root and shoot proteins are shown in Supplemental Figures S7 and S8. Numbers of significantly changing (*x*) and total quantified (*y*) proteins for each subcellular location (*x/y*) are shown. A comparison of k-samples distributions (Kruskal–Wallis) and multiple pairwise comparisons using the Conover–Iman procedure were performed in XLSTAT to evaluate the level of changes in subcellular locations. Changes in abundance of proteins in root can be divided into A–G groups with increasing values. Changes in abundance of proteins in shoot can be only divided into A group.

To dissect the role of autophagy in protein homeostasis in different cellular compartments, we displayed the distributions of relative change in protein abundance according to the known subcellular localization of each protein (Hooper et al., 2014, 2017; Figure 1;  Supplemental Data Set 2). Proteins located in the cytosol and peroxisomes of both shoots and roots showed higher median abundance in *atg11* and *atg5* mutants than in *Wt*. Conversely, proteins in the nucleus, plasma membrane, vacuoles, and those secreted to the extracellular space, showed lower median abundances in the autophagy mutants compared to *Wt*. The majority of the 53 plastid proteins found in roots showed lower abundance in the mutants, but an overall increase in chloroplast protein abundance was observed in shoot tissue from both mutant lines (Figure 1). A higher abundance of mitochondrial proteins was found in both shoots and roots of *atg11*, but only in the shoot of *atg5* compared to *Wt*.

To further understand the role of autophagy in regulating the protein synthesis and degradation machineries, we investigated abundance changes in ribosomal and proteasomal subunits in both mutants compared to *Wt* (Supplemental Figure S6; Supplemental Data Set 2). For ribosomes, 70%–80% of r-subunits showed a trend of higher abundance in the mutants, with 17 out of 61 r-protein in root and 2 out of 7 ribosomal r-proteins in shoot showing statistically significant increases (Student’s *t* test, *P* < 0.05). More than half of the proteasomal subunit proteins identified also tended to be more abundant in both mutants, with 2 out of 23 proteasomal subunit proteins in root and 1 out of 6 in shoot showing statistically significant increases (Student’s t test, *P* < 0.05).

### Specific proteins changed in abundance in autophagy-deficient plants

To investigate specific protein abundance changes in root (Supplemental Figure S7) and shoot (Supplemental Figure S8), proteins with statistically significant changes in *atg5* and *atg11* were then categorized by their subcellular localizations and functions.

In roots, the cytosolic Chaperonin Containing T-complex polypeptide-1 (CCT) protein complex subunits, ribosomal subunits, enzymes of amino acid metabolism (GAD1, ASP2, MMT, and OLD3), and glycolytic enzymes accumulated in *atg11* and *atg5* (Supplemental Figure S7). In contrast, cytoskeleton-related proteins including villins (VLN4), actin (ACT7), and tubulin (TUB2, 4, 6, 8, and 9), enzymes of amino acid metabolism (MAT3 and BCAT4), and phosphatidylinositol transfer proteins (At1g30690 and At1g72160) from the secretory pathway showed decreased abundance in both *atg11* and *atg5*. Eleven mitochondrial proteins, including components of the electron transport chain and TCA cycle, showed increases in abundance; several mitochondrial stress response proteins, such as mtHsc70-1, mtHsc70-2, and GPX6, displayed a decreased abundance in both mutants. Ten mitochondrial proteins (including the ATP synthase beta subunit, CPN10, ATPHB3, TOM5, and carbonic anhydrase) showed different patterns in *atg11* and *atg5*, with their abundance typically increased in *atg11* but decreased in *atg5* (Supplemental Figure S7). We also found that in roots, proteins involved in vesicle transport specifically accumulated in *atg5* but not in *atg11*; these proteins included clathrin heavy chain1 (At3g11130) associated with plasma membrane and Golgi, and the coatomer alpha, delta, and gamma-subunits (At2g21390, At5g05010, and At4g34450) of the COP1 coat, which is required for intra-Golgi transport, retrograde transport from Golgi to ER, and Golgi maintenance. ER-resident proteins, such as AtBAG7(At5g62390), CNX1 (At5g61790), and PDIL1-3 (At3g54960), also show a higher abundance in *atg5* than *atg11* when compared to *Wt* (31% in *atg5* versus 6% in *atg11*).

Different sets of proteins were quantified in shoots compared with roots due to the variation in their absolute abundance in photosynthetic and nonphotosynthetic tissues. In shoots, almost half of quantified cytosolic proteins with significant changes in abundances were less abundant in mutant lines (Supplemental Figure S8). Similar to the protein set from the roots, cytosolic ribosomal subunits, enzymes of amino acid metabolism (methionine adenosyltransferase 3-MAT3 and cobalamin-independent synthase-ATCIMS) and glycolytic enzymes from shoots showed increased abundance, while profilin1 and profilin2, which regulate the organization of actin cytoskeleton, showed reduced abundance in both *atg11* and *atg5*. Peptidylprolyl isomerase enzymes (FK506-binding protein 12-FKBP12, rotamase cyclophilin 1,3 and 5-ROC1, ROC3, and ROC5) and proteins with redox activity (thioredoxin 3-TRX3, thioredoxin-dependent peroxidase 1-TPX1, and copper/zinc superoxide dismutase 1-CSD1) showed decreased abundance in both *atg11* and *atg5* shoots. In shoots, the mitochondrial redox proteins (glutathione peroxidase 6-GPX6 and peroxiredoxin IIF-PRXIIF), CPN10, and membrane-localized electron transport chain subunits showed decreased abundance while TCA cycle enzymes and matrix-localized ETC subunits accumulated in both *atg11* and *atg5*. In chloroplasts, most stromal proteins showed increased abundance, while PSII subunits, PSI reaction center (PSAN), cytochrome *b_6_/f* (PetC), plastocyanin (DRT112 and PETE1), thioredoxins, and protein folding-associated proteins were less abundant in both *atg11* and *atg5* (Supplemental Figure S8). Most quantified shoot plastid proteins showed consistent changes in abundance in both mutant lines with few exceptions.

**Figure 2:**
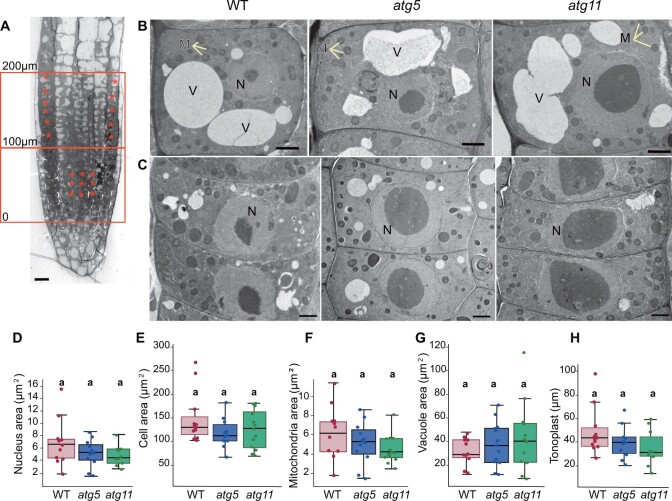
Transmission electron microscopy analysis of *Wt*, *atg5*, and *atg11* root cells. A, Longitudinal section of a *Wt* root showing the areas selected for analysis: meristem region (up to 100 µm from QC) and the adjacent area up to 200 µm from the QC where cells are actively developing vacuoles. Asterisks indicate examples of cells that were analyzed. B, Cell with developing vacuoles (C) Meristematic cells; (D and –E) Quantification of vacuolated cell area per section (D), nuclear area in meristematic cells (E), mitochondrial and vacuolar area per section of vacuolated cells (F, G) and length of tonoplast per section of vacuolated cells (H). Between 10 and 13 cells from two roots of each genotype were used for this analysis with the ANOVA post-hoc Tukey’s test for grouping. M, mitochondria; N, nucleus; V, vacuole. Scales bars = 10 µm in (A); 2 µm in (B and C). Box plots indicate the median (center lines), interquartile range (borders of boxes) and minimum and maximum values (whiskers).

To determine whether these many changes in the abundance of specific root proteins were reflected in changes in the cellular architecture, we analyzed the ultrastructure of root tips of 24-day-old *Wt*, *atg5*, and *atg11* plants processed by high-pressure freezing/freeze substitution and resin-embedding. In longitudinal sections of root tips, we identified two areas of interest, the meristematic area (up to 100 microns from the quiescent center (QC) toward the elongation zone) and the area where cells started to develop large vacuoles (between 100 and 200 microns from the QC) (Figure 2, A–C). We imaged multiple middle sections of two roots of each genotype and measured the cell area and the area occupied by the nucleus, mitochondria, and vacuoles as well as the tonoplast length per section (Figure 2, D–H). We did not find statistically significant differences in any of these parameters between *Wt* and *atg* mutants; however, there were consistent trends showing slight increase in vacuole surface and a reduction in tonoplast length/perimeter in the two *atg* mutants, in actively vacuolating cells. These results indicate that the changes in the proteome of *atg5* and *atg11*, did not induce drastic changes in the cellular organization of mutant root cells.

However, we noticed that ∼25% of the cells in the *atg5* root tips contained abnormal Trans-Golgi networks (TGNs) with largely dilated bulges or vesicles (Supplemental Figure S9, A and B) and large concentric membranous systems (Supplemental Figure S9, C–F). In some cases, the edges of these abnormal large membranes had bulges and budding profiles reminiscent of Golgi/TGN cisternae (Supplemental Figure S9, C and D). In some other examples, we were able to image coats assembled on budding sites on the membrane edges (Supplemental Figure S9E, arrowheads), which is consistent with the abnormal accumulation of COP1 coatomer subunits and clathrin in *atg5*. Whereas most of these structures seem to enclose ribosomes and cytoplasm, ∼10% of them displayed rounded electron dense aggregates 2–3 times larger than a ribosome (Supplemental Figure S9E).

### Autophagy deficiency changes the degradation rate of specific organelle proteins in Arabidopsis root and shoot

To compare specific protein abundance changes with changes of specific protein degradation rates, we utilized a ^15^N progressive labeling strategy (Li et al., 2017) to quantify protein degradation rates in the three genotypes. For this, the media of hydroponically grown plants was switched from ^14^N to ^15^N nutrient salts to label newly synthesized proteins over 3 days, and the fraction of each peptide that was ^15^N-labeled (Labeled Peptide Fraction [LPF]) was calculated using peptide MS. In total, LPF for 11,179 peptides in roots and 7,145 peptides in shoots was quantified in three biological replicates across the three genotypes. From these LPFs, the degradation rates (*K*_D_, d^−1^) of 558 root proteins and 505 shoot proteins with high abundance in plant cells were obtained (Supplemental Data Set 3) and relative changes in *K*_D_ values were visualized by volcano plots (Supplemental Figure S10). In roots, most proteins with slower degradation rates in *atg11* (68%) and *atg5* (82%) were located in the cytosol, followed by smaller proportions that were located in mitochondria and ER (Table 1). In shoots, proteins that degraded slowly were predominantly from the cytosol, chloroplasts, and mitochondria. A higher proportion of mitochondrial proteins with slower degradation rates were detected in *atg11* roots and shoots (17% and 21%) compared to *atg5* (2% and 0%). There was also a higher proportion of chloroplastic proteins with slower degradation rates in shoots of *atg11* (33%) compared to shoots of *atg5* (7%).

**Table 1:** Cellular compartments with significant slower protein degradation rate in *atg5/11* mutants compared with *Wt* Arabidopsis

Tissue	*atg11/Wt*, % (*n/N*)	*atg5/Wt*, % (*n/N*)	Subcellular Location
Root	68 (45/66)	82 (42/51)	Cytosol
	17 (11/66)	2 (1/51)	Mitochondrion
	5 (3/66)	4 (2/51)	ER
	11 (7/66)	12 (6/51)	Others
Shoot	33 (8/24)	64 (9/14)	Cytosol
	33 (8/24)	7 (1/14)	Chloroplast
	21 (5/24)	0 (0/14)	Mitochondrion
	13 (3/24)	29 (3/14)	Others

Percentage of proteins resident in major cellular compartments that showed a significantly slower degradation rate in *atg11* and *atg5* than in *Wt*. In root, the majority of proteins with slower degradation rate are located in the cytosol, mitochondrion, or ER. In shoot, the majority are in the cytosol, chloroplast, and mitochondrion. Cellular localization of proteins beyond the top three are marked as others. Numbers in brackets are the number of proteins with slower protein degradation rate divided by the total number of proteins from that location that were analyzed.

In roots, proteins with significantly slower degradation rate in both *atg5* and *atg11* (Supplemental Figure S11) included forty cytosolic proteins, two ER proteins (the chaperones calreticulin 1a-CRT1 and calnexin 1-CNX1), and one mitochondrial protein (ATP synthase D chain). Cytosolic proteins in this list can be broadly placed into three major functional categories: metabolism, ribosome subunits, and glycolytic enzymes. In shoots, 12 cytosolic proteins showed slower degradation rates in both *atg5* and *atg11*. We also found proteins with slower rates of protein degradation but with statistical significance only in one of the two mutants (Supplemental Data Set 3). One example from this group was RPN10, which has been reported to be an autophagy receptor for the proteasome (Marshall et al., 2015). In contrast, four mitochondrial and eight chloroplastic proteins showed very different changes in degradation rate between *atg5* and *atg11*. These proteins show slower degradation rate in *atg11*, but no change or faster rates of degradation in *atg5*. These patterns suggested a specialized role of ATG11 in mitochondrial and chloroplast protein homeostasis.

### Identification of ATG5 and ATG11 targets from the combined protein degradation rate and abundance data

**Figure 3:**
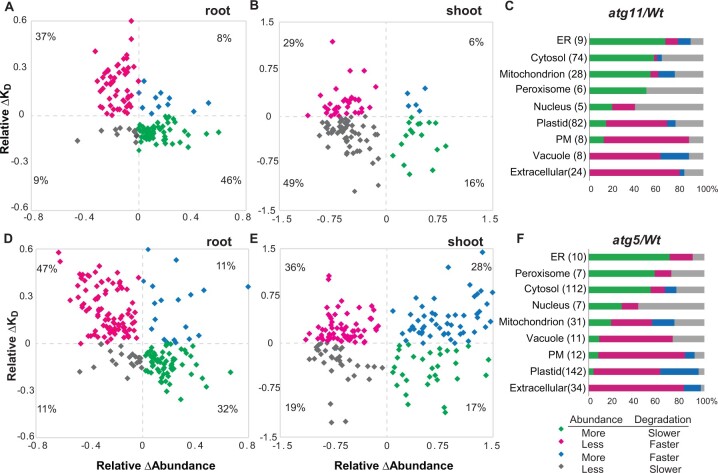
Combination of changes in abundance and degradation rate for proteins in *atg5 and atg11* from different cellular compartments. Matching sets of protein degradation rate changes (Relative Δ*K*_D_) and protein abundance changes (Relative ΔAbundance) were graphed orthogonally to identify putative autophagy cargo. About 140 root proteins and 116 shoot proteins with significant changes in abundance or degradation (Student’s *t* test, *P* < 0.05) in *atg11* are plotted in (A) and (B). About 200 root proteins and 187 shoot proteins with significant changes in abundance/degradation (Student’s *t* test, *P* < 0.05) in *atg5* are plotted in (D) and (E). The combined proportions of root and shoot proteins shown in each quadrant that reside in a particular subcellular location are shown in (C) and (F) for *atg11* and *atg5* separately. The four colors represent the four quadrants. Group 1 represents proteins with slower degradation rates and greater abundance, consistent with direct changes driven by deficient autophagy substrate degradation; Group 2 represents proteins with faster degradation rates and a lower steady-state abundance, potentially driven by alternative degradation pathways compensating for defects in autophagy; Group 3 contains proteins with faster degradation rates and greater abundance (likely driven by enhanced protein synthesis); and Group 4 contains proteins with slower degradation rates and lower abundance, putative examples of feedback regulated response to impaired autophagy degradation triggering decreasing protein synthesis.

Protein abundance and degradation rate changes were then plotted orthogonally to pinpoint probable autophagy protein targets (Figure 3;  Supplemental Data Set 4). When *atg11* and *atg5* were compared to *Wt*, we found that 140 and 200 root proteins and 116 and 187 shoot proteins showed significant changes in abundance and/or degradation rate (Student’s *t* test, *P* < 0.05). The response of these proteins could be grouped into the four quadrants with different responses as explained in Figure 3.

**Figure 4:**
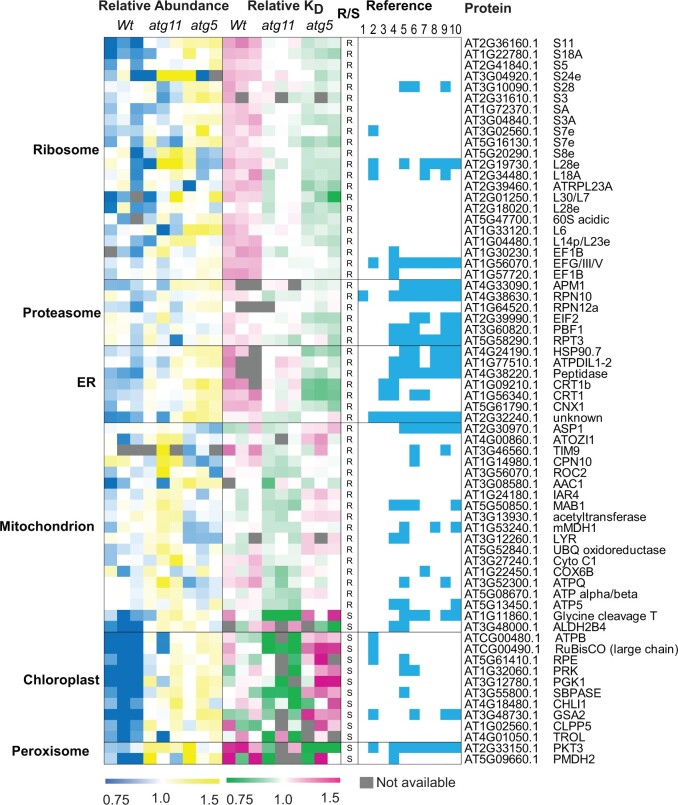
Identification of known autophagy targets in Arabidopsis roots (R) and shoots (S) as proteins with higher abundance and slower degradation rates in *atg5 or atg11* compared with *Wt*. Sixty-six proteins with significant differences in the abundance or degradation rate (Student’s *t* test, *P* < 0.05) in *atg11* or *atg5* are shown in heatmaps. Relative protein abundance/degradation values were normalized to median value in all nine samples (Supplemental Data Set 6). Normalized relative abundance/degradation values are shown by colour gradients. Proteins are grouped according to their function in the ribosome, proteasome, or chaperones or their location in the ER, mitochondrion, chloroplast, or peroxisome. Reference columns show the presence-cyan/absence-white of reported autophagy targets from six independent literature reports. Column 1 was acquired from comparison with Arabidopsis ATG8 interactors (Marshall et al., 2019), Column 2 with Arabidopsis homologs in potato (*Solanum tuberosum*; Zess et al., 2019), Column 3 with iLIR data for Arabidopsis (Kalvari et al., 2014), Column 4 was acquired from comparison to an *atg5* mutant line (under control, low nitrogen, and sulfur conditions; Have et al., 2019), Columns 5–8 from Arabidopsis homologs in maize leaves plus or minus nitrogen limitation (McLoughlin et al., 2018), Columns 9 and 10 from Arabidopsis homologs in maize leaves plus or minus carbon limitation (McLoughlin et al., 2020).

**Figure 5:**
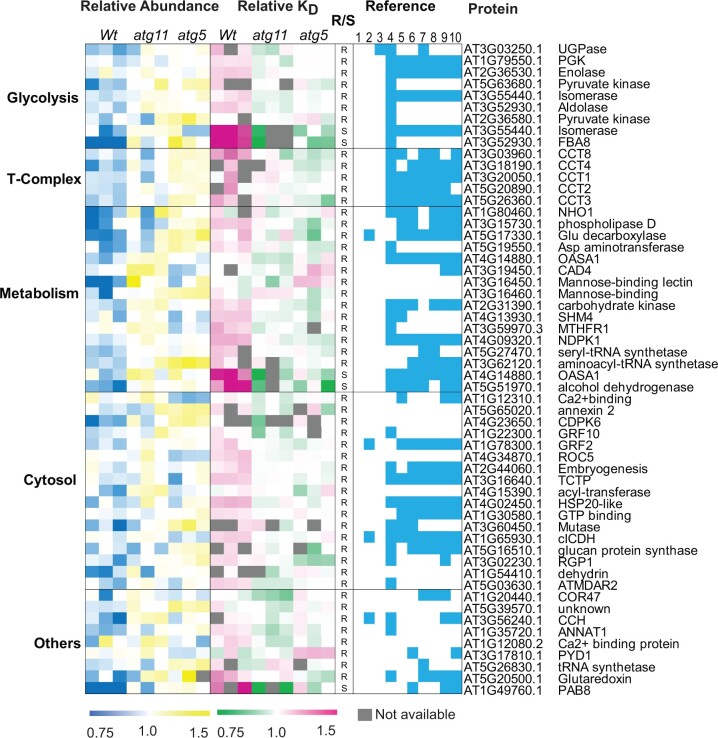
Identification of putative ATG5 and ATG11 targets in Arabidopsis roots (R) and shoots (S) as proteins with higher abundance and slower degradation rates in *atg5 or atg11* compared with *Wt*. Fifty-six proteins with significant differences in the abundance or degradation rate (Student’s t test, *P* < 0.05) in *atg11* or *atg5* are shown in heatmaps. Normalized relative protein abundance/degradation values were shown as mentioned in Figure 4. Proteins grouped according to their functions in glycolysis, T-complex, metabolism, cytosol, and others. Reference columns show the presence/absence of reports as putative autophagy targets from the six independent reports mentioned in Figure 4.

Of the significantly changed proteins in roots, 80% were more abundant and slow degrading (Group 1) or less abundant and faster degrading (Group 2) (Figure 3, A and D). The former are potential autophagy targets. These proteins are typically localized to the ER, cytosol, peroxisome, nucleus, and mitochondrion (Supplemental Data Set 5). Furthermore, they are components of mitochondrial oxidative phosphorylation, amino acid metabolism, glycolysis, the ribosome and proteasome, TCP-1 chaperones, and protein folding and processing in the ER (Figures 4 and 5). In comparison, most of the proteins that were degraded faster but accumulated less in the mutants (Group 2) were potential components of alternative and/or compensatory pathways and were localized to vacuoles, plasma membrane, plastids, and apoplast (Supplemental Data Set 5). From these, it was apparent that the mitochondrial TCA cycle and oxidative phosphorylation proteins showed the most distinct differences between the mutants, with 17 mitochondrial proteins belonging to Group 1 in *atg11* but not in *atg5* (Figure 3C;  Supplemental Data Set 5*)*.

In shoots, only 50% of proteins that significantly changed were in Groups 1 and 2, and Group 1 accounted for ˂20% of proteins in both mutants (Figure 3, B and E). A high proportion of proteins from shoots in Group 2 localize to plastid, vacuole, or the apoplast (Supplemental Data Set 5). The higher proportion of proteins that fell into Group 2 in shoots compared to roots might suggest that protein synthesis attenuation masks autophagy degradation in shoot tissues. In *atg11*, most of the remaining proteins were in Group 4 while in *atg5*, a higher proportion of proteins were in Group 3. Group 1 from shoots included mainly resident peroxisome, cytosolic, mitochondrial, and chloroplastic proteins, with a smaller proportion of nuclear and vacuolar proteins (Supplemental Data Set 5). Proteins in the chloroplast showed different responses between mutant lines; 10 chloroplastic proteins, including RUBISCO large subunit, fell into Group 1 in *atg11* but in Group 3 in *atg5* (Supplemental Data Set 5). More chloroplastic and mitochondrial proteins with slower turnover rate were detected in *atg11* than *atg5* when root and shoot data were combined (Figure 3, C and F). This again was consistent with reports of ATG11 playing a specialized role in mitochondria and chloroplast protein degradation.

**Figure 6:**
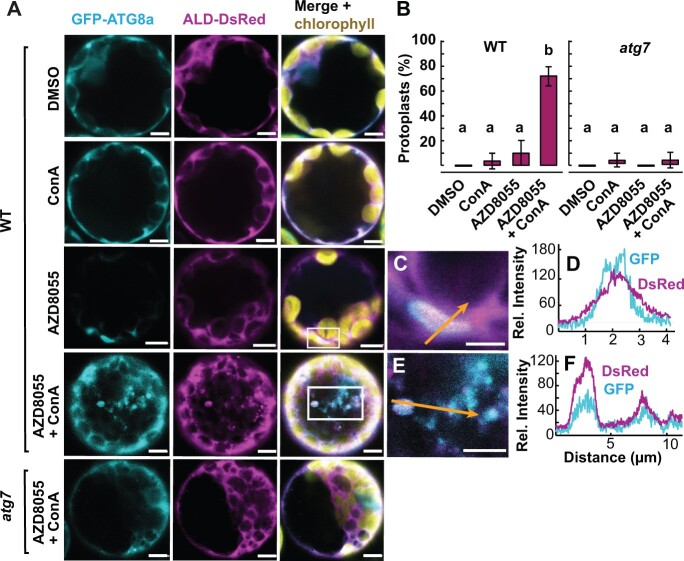
Autophagy participates in degradation of glycolytic enzyme FBA8 in Arabidopsis protoplasts. Representative Arabidopsis protoplasts co-expressing GFP-ATG8a and FBA8-DsRed treated with DMSO (control), 0.5-μM ConA, and/or 0.5-μM AZD8055 in *Wt* and *atg7* mutant (A). B, Percentage of observed protoplasts showing vacuolar autophagy bodies positive for both GFP-ATG8a and FBA8-DsRed. This graph shows the averages of three independent biological replicates (±sd) with the ANOVA post-hoc Tukey’s test for grouping; *n* = 24–30 protoplasts for each treatment. C and E, Enlarged images of the areas highlighted by white boxes in the preceding confocal images. D and F, Fluorescence intensity profiles of GFP-ATG8a and FBA8-DsRed along the arrows in (C) and (E), respectively. Scale bars = 5 µm in (A) and (E); 2 µm in (C).

In total, 122 potential targets of autophagy were identified by combined abundance and degradation analysis in root and shoot tissues of *atg5* or *atg11* (Figures 4 and 5;  Supplemental Data 6). Several lines of evidence support them as selective targets of autophagy in plants. For example, 71 of them have been predicted or reported to be ATG8 interactors or shown to accumulate in autophagy mutants (Supplemental Data Set 6; Kalvari et al., 2014; McLoughlin et al., 2018, 2020; Have et al., 2019; Marshall et al., 2019; Zess et al., 2019). Sixty-six proteins are in complexes or organelles shown to be autophagy targets as mentioned in the introduction, namely ribosome, proteasome, ER, mitochondrion, chloroplast, and peroxisome (Figure 4). We propose 56 proteins as putative autophagy protein targets as, to our knowledge, they have not been evidenced by autophagy-based degradation before (Figure 5). Nine glycolytic enzymes were among this putative protein list. FBA8 was chosen as a case to validate it as a genuine autophagy cargo. We found that FBA8 can be internalized into autophagosomes during autophagy stimulated by a TOR kinase inhibitor AZD8055 in Arabidopsis protoplasts (Figure 6). However, no FBA8 can be internalized into autophagosomes in Arabidopsis protoplasts prepared from *atg7* leaves. Furthermore, FBA8 accumulated in mutant lines and its degradation in *Wt* Arabidopsis plants was inhibited by cysteine protease inhibitor E64d and vacuolar acidification inhibitor concanamycin (ConA), both of which are attenuators of autophagy-associated vacuolar degradation (Supplemental Figure S12).

### Pi limitation induces autophagy and changes metabolite abundances in hydroponically grown Arabidopsis

Nitrogen, Pi, or carbon limitation are reported to activate autophagy, promote cellular content degradation in plants, and lead to early senescence in autophagy mutants (Yoshimoto et al., 2009b; Avin-Wittenberg et al., 2015; Barros et al., 2017; McLoughlin et al., 2018, 2020; Have et al., 2019; Naumann et al., 2019). However, it is unclear if such conditions lead to a generic induction of autophagy or of selective autophagy of stress-related targets. Nitrogen limitation conditions would limit our ability to use ^15^N labeling and darkness would limit both carbon and ^15^N incorporation into amino acids (Nelson et al., 2014). Therefore, we subjected plants to Pi starvation to investigate its effect on protein abundance and degradation rate in all three genotypes (Supplemental Figure S13).

No visible phenotypic changes were observed in plants grown under Pi-limited conditions over 3 days of treatment (Supplemental Figure S13A), although both root and shoot Pi content was significantly reduced in all genotypes (Supplemental Figure S13B). To monitor autophagy induction and autophagic flux, we performed an imaging analysis of a line expressing *GFP-ATG8a*, which is localized to autophagic membranes and autophagosomes in root cells. Abundant GFP-ATG8a-decorated organelles were evident in the elongation zone of Pi-limited roots but not in the equivalent root zone from control plants (Supplemental Figure S13C). In shoots, the *Fv/Fm* ratio remained at 0.8 in all three genotypes under control and Pi-limiting conditions (Supplemental Figure S13D). Consistent with the reduced Pi content, the transcript of the Pi sensor *SPX1* was induced in all three genotypes when plants were grown under Pi-limiting conditions (Supplemental Figure S13E). Autophagy-associated genes, *ATG8H* and *ATG7*, were also induced under limited Pi in *Wt* and *atg5* but not in *atg11* plants (Supplemental Figure S13E). Extension of Pi-limited conditions to >10 days led to purple coloration of rosette leaves, indicating stress-induced anthocyanin accumulation.

**Figure 7:**
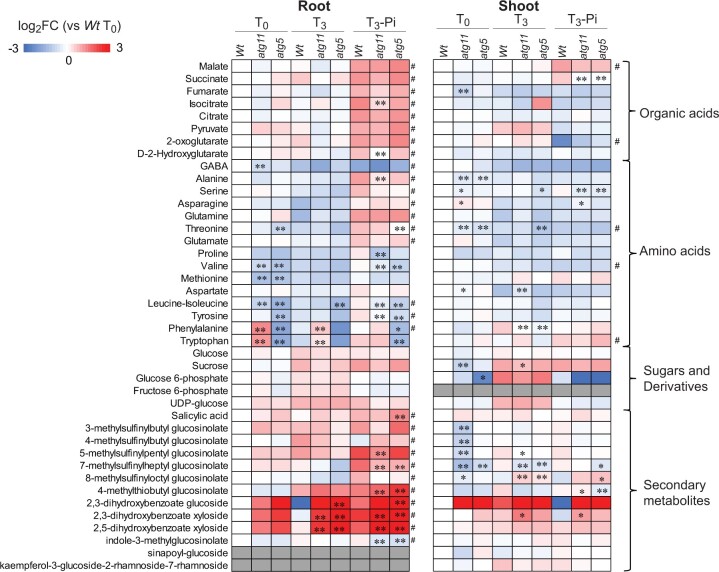
Primary and secondary metabolite profile changes under control conditions and Pi limitation in *atg5* and *atg11*. Heatmaps of changes in metabolite abundance in shoots and roots are shown. Metabolites were determined by LC/MS. Two-way ANOVA analyses were carried out to determine the genotype and Pi starvation effects on metabolite abundance changes. Metabolite content significantly altered due to Pi starvation, regardless of genotype, was labeled by a hashtag (#) (*P* < 0.05 for both T0 versus T3−P and T3−P versus T3 + P). Metabolite content significantly altered due to genotype was determined by Tukey Ad hoc analysis for Col-0 versus either *atg5* or *atg11* and labeled by an asterisk; *P < 0.05 or **P < 0.01. Metabolites with unquantifiable abundance in a given sample are shown in gray.

To further assess the impact of Pi limitation on metabolism, we profiled the abundance of selected primary and secondary metabolites in shoots and roots both between *Wt* and autophagy mutants and within each genotype (Figure 7;  Supplemental Data Set 11). Pi limitation led to the general accumulation of organic acids, amino acids, and secondary metabolites in roots in all three lines, but only accumulation of malate and tryptophan in shoot tissues.

Previously in roots, a drastic decrease of amino acids is reported in response to nutrient limitation conditions (Araújo et al., 2011; Masclaux-Daubresse et al., 2014; Avin-Wittenberg et al., 2015; Barros et al., 2017). However, we observed very little effect on the abundance of amino acids and sugars in both mutants upon Pi limitation. Organic acids in roots generally did not change in abundance except for isocitrate and D-2-hydroxyglutarate, which were slightly more abundant in *atg11* under Pi starvation. Several glucosinolates changed in abundance in roots of autophagy mutants only under Pi limitation. Salicylic acid (SA) levels did not change in mutants under control conditions but accumulated in *atg5* under limited Pi (Figure 7), as also previously reported in dark-induced senescence (Yoshimoto et al., 2009b). Interestingly, SA–sugar conjugates, including 2,3-dihydroxybenzoate glucoside 2,3-dihydroxybenzoate xyloside, and 2,5-dihydroxybenzoate xyloside, accumulated in autophagy mutants under both control and Pi-limiting conditions. SA conjugation inactivates SA; the accumulation of these compounds in autophagy mutants might be part of a mechanism to partially prevent the SA-dependent early senescence typical of autophagy mutants (Yoshimoto et al., 2009b).

In shoots, there was even less metabolite response to Pi limitation than in roots. However, Pi limitation did increase succinate level in shoots of *Wt* but not mutants. Pi limitation did not further decrease the levels of alanine, threonine, serine, and phenylalanine, which were already less abundant in the mutant lines under control conditions. Moreover, asparagine and fumarate showed specific changes in abundance in the two mutant lines. Asparagine showed some accumulation specifically in *atg11* but not in *atg5* under both control and Pi-limiting conditions. Fumarate was reduced in *atg11* only under control conditions. Few changes in sugar and sugar derivative abundances were present in shoots, with glucose and glucose-6-phosphate decreasing slightly in *atg11* or *atg5*, respectively. All glucosinolates, except 8-methylsulfinyloctyl glucosinolate, decreased in abundance in shoots of autophagy mutants. Consistent with the patterns seen in roots, SA–sugar conjugates in shoots were more abundant in autophagy mutants, but this accumulation was only statistically significant for 2,3-DHBX in *atg11*.

Overall, these profiles indicate that many of the metabolic effects of autophagy disruption are already evident under control conditions, while some of them were enhanced by Pi limitation.

### Pi limitation had only a mild impact on root cytosolic protein degradation in mutant lines and on mitochondria abundance in *atg11*

**Figure 8:**
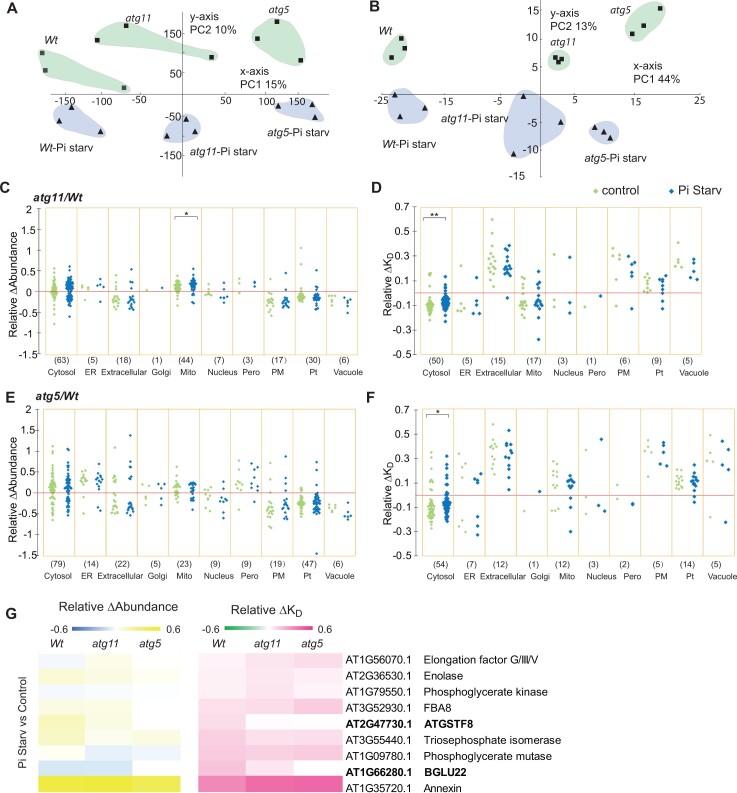
Pi limitation effects on changes of root protein abundance and degradation in *atg5* and *atg11*. A and B, PCA was applied to evaluate Pi limitation effects on protein abundance and degradation changes in *Wt*, *atg11*, and *atg*5 using 1,045 and 476 proteins, respectively. Protein abundance data were LN transformed before being used for PCA. Principle components 1 and 2 (*x-* and *y*-axes) for all genotypes under both control and Pi starvation conditions are shown for protein abundance (A) and protein degradation (B). Relative changes of protein abundance and degradation between *Wt* and autophagy mutant lines were plotted to visualize Pi limitation effects on specific proteins of known location in root cells. Relative changes in protein abundance from 194 proteins in *atg11/Wt* comparisons (C) and from 233 proteins in a*tg5/Wt* comparisons (E) are shown as scattergrams. Relative changes in protein degradation rates from 111 proteins in *atg11/Wt* comparisons (D) and from 115 proteins in a*tg5/Wt* comparisons (F) are also shown as scattergrams. A nonparametric Kolmogorov–Smirno test was utilized for comparison of control and Pi starvation on distribution of relative changes in protein abundance and degradation rate of cellular localizations to evaluate the Pi limitation effect (**P < 0.01, *P < 0.05). Nine proteins show significantly faster degradation rates under Pi starvation conditions compared with control in *Wt* root. Relative degradation rate changes (relative Δ*K*_D_) and relative abundance changes (relative Δabundance) of these proteins in *Wt*, *atg5* and *atg11* between control and Pi limiting conditions are shown as heatmaps (G). Proteins with slower degradation rate in mutant lines are shown in bold font.

To determine if protein degradation rates were similarly affected by Pi limitation, we compared protein abundance for 1,045 proteins and degradation rates for 476 proteins among *Wt*, *atg5*, and *atg11* roots under both control and Pi-limiting conditions. A principal component analysis (PCA) of these datasets showed that each genotype/treatment group could be separated by protein abundance and degradation rate (Figure 8, A and B; Supplemental Figure S14). Low Pi increased the abundance of vacuole proteins and decreased the abundance of Golgi proteins in *Wt*; however, the same treatment caused an increase in vacuolar proteins in *atg11* but not in *atg5*, whereas Golgi proteins were not significantly altered in either mutant (Supplemental Figure S15). Low Pi did not induce significant changes in protein degradation rates in *Wt*; however, it did decrease mitochondrial protein degradation rates in both *atg11* and *atg5*, and peroxisomal protein degradation rates in *atg5* (Supplemental Figure S16).

We then expressed the root datasets as relative changes in mutants and compared them between control and Pi-limiting conditions (Figure 8, C–F). We found that mild Pi limitation further increased mitochondrial protein abundance in *atg11* compared to *Wt*, but not in *atg5*. Unexpectedly, Pi limitation decreased the degree of differences in degradation rates of cytosolic proteins between the mutant lines and *Wt* (Figure 8, D and F). This is seen in the narrower distribution of relative ΔK_D_ values under Pi limited conditions. However, we found nine proteins, including five cytosolic glycolytic enzymes, Annexin 1, the glutathione transferase ATGSTF8 and the ER-localized beta-glucosylase BGLU22, with significantly faster degradation rates under Pi limitation in *Wt* (Figure 8G;  Supplemental Data Set 10). Intriguingly, the faster degradation of these proteins under low Pi did not lead to a decrease in their abundance; rather, four out of nine proteins were more abundant under low Pi conditions. This pattern is consistent with induced protein synthesis as a means to compensate for faster protein degradation under Pi limitation. Faster degradation of AtGSTF8 and BGLU22 under low Pi were only detected in *Wt* but not in the autophagy mutants (Figure 8G;  Supplemental Data Set 10).

### Pi limitation affects the degree of relative changes in abundance of chloroplast proteins in shoots and their degradation rates in both *atg5* and *agt11*

**Figure 9:**
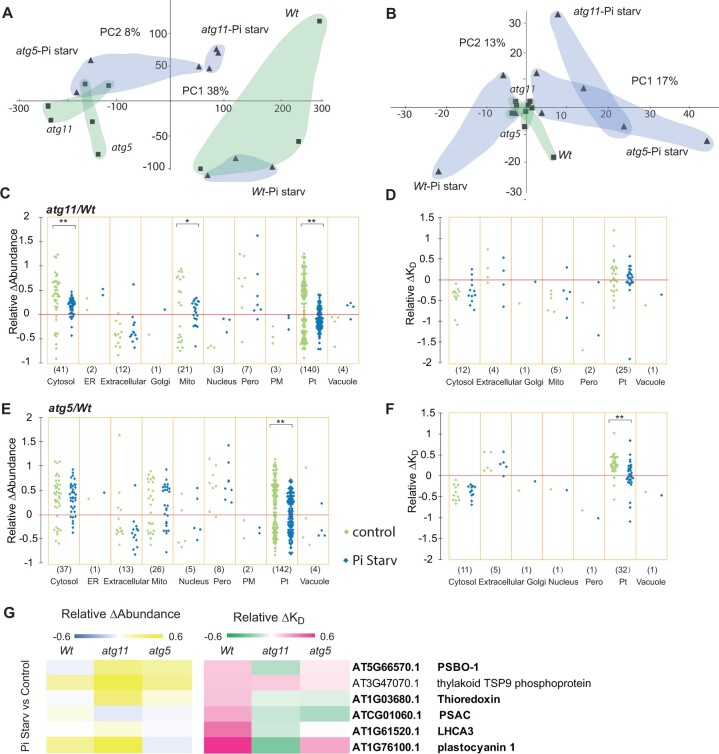
Pi limitation effects on changes of shoot protein abundance and degradation in *atg5* and *atg11*. A and B, PCA was applied to evaluate Pi limitation effects on protein abundance and degradation changes in *Wt*, *atg11* and *atg*5 using 782 and 505 proteins, respectively. Protein abundance data were LN transformed before being used for PCA. Principle components 1 and 2 (x and y axis) for all genotypes under both control and Pi starvation conditions are shown for protein abundance (A) and protein degradation (B). Relative changes of protein abundance and degradation between *Wt* and autophagy mutant lines were plotted to visualize Pi limitation effects on specific proteins of known location in shoot cells. Relative changes in protein abundance from 238 proteins in *atg11/Wt* comparisons (C) and from 234 proteins in a*tg5/Wt* comparisons (E) are shown as scattergrams. Relative changes in protein degradation rates from 50 proteins in *atg11/Wt* comparisons (D) and from 52 proteins in a*tg5/Wt* comparisons (F) are also shown as scattergrams. Two-sample Kolmogorov–Smirno test (**P < 0.01, *P < 0.05) was utilized for comparison of control and Pi starvation on distribution of relative changes in protein abundance and degradation rate of cellular localizations to evaluate the Pi limitation effect. Six proteins show significantly faster degradation rates under Pi limiting conditions compared with control in *Wt* shoot. Their relative degradation rate changes (relative Δ*K*_D_) and relative abundance changes (relative Δabundance) in *Wt*, *atg5*, and *atg11* between control and Pi limiting conditions are shown as heatmaps (G). Proteins with slower degradation rate in mutant lines are shown in bold font.

We also compared protein abundance of 782 proteins and degradation rates of 505 proteins among *Wt*, *atg5*, and *atg11* shoots under control and Pi-limiting conditions. By applying a PCA, we found that protein abundance in *Wt* samples could be separated from *atg5* and *atg11* under both control and Pi-limiting conditions, while *atg5* and *atg11* samples can be clearly separated under Pi limitation but not under control conditions (Figure 9A;  Supplemental Figure S14). In terms of protein degradation rates, *Wt* samples could be fully separated by PCA from *atg5* and *atg11* under low Pi, but not under control conditions (Figure 9B). Pi limitation led to a decrease in cytosolic protein abundance in *Wt* and *atg11* but not in *atg5.* Chloroplastic proteins accumulated in *Wt* under low Pi whereas under similar conditions, chloroplastic protein abundances decreased in both mutant lines (Supplemental Figure S15). Pi limitation conditions altered protein degradation rates of chloroplastic proteins only in *atg5* (Supplemental Figure S16).

As done for the root datasets, we then expressed the shoot data as relative changes in mutants to facilitate comparisons between samples grown under control and Pi-limiting conditions (Figure 9, C–F). Low Pi again led to a narrower distribution of the abundance changes of chloroplastic proteins in both mutant lines compared to *Wt* and smaller changes of protein degradation rate for chloroplast proteins in *atg5.* Conversely in *atg11*, Pi limitation was associated with a narrower distribution of changes in cytosolic and mitochondrial protein abundance without affecting the changes in protein degradation rate. Although there was no overall change in organellar degradation rate, six shoot proteins (PSBO-1, thylakoid TSP9 phosphoprotein-At3g47070, thioredoxin-At1g03680, PSAC, LHCA3, and plastocyanin 1) showed significantly faster degradation rates under Pi limiting conditions in *Wt* (Figure 9G;  Supplemental Data Set 10). Four of them, namely PSBO-1 (PSII), thioredoxin (chloroplast stroma), PSAC (PSI), and LHCA3 (PSI), showed unchanged or slower degradation rate in *atg11* and *atg5* under low Pi. These are, therefore, potential ATG11- and ATG5-associated targets of induced autophagy under low Pi. Plastocyanin 1 (thylakoid) showed a slower degradation rate and significant increase in abundance in *atg11* but faster degradation in *atg5* and nonsignificant change in abundance, which suggests its degradation is dependent on ATG11 but not ATG5. A notable exception to these trends was the thylakoid TSP9 phosphoprotein, which is involved in photosystem state transition (Fristedt et al., 2009), that had an increased degradation rate but also accumulated in abundance in *Wt* and mutant lines under low Pi, indicating its high protein synthesis rate and its turnover in all lines under Pi limitation.

## Discussion

### Investigation of protein turnover in autophagy mutant lines

Defects in autophagy can cause accumulation of autophagic protein cargo due to impaired degradation, but also lead to many changes in metabolite and transcript abundances, complicating the interpretation of cause and effect. Increase in transcript abundance may reveal upregulation of gene expression (McLoughlin et al., 2018, 2020), but that does not necessarily correlate with protein synthesis. In addition, estimating protein synthesis by ribosome profiling (Juntawong et al., 2014; Chotewutmontri and Barkan, 2016) or newly made protein labeling strategies (Wang et al., 2016) in autophagy mutants can be misleading since ribosomes themselves are targets of autophagy (Gretzmeier et al., 2017; McLoughlin et al., 2018; Figure 4;  Supplemental Figure S7). Focusing on steady-state protein abundance alone in impaired autophagy mutants also fails to identify proteins that may maintain homeostasis, either by using alternative degradative pathways or reducing protein synthesis. By focusing on protein degradation rates and correlating these with protein abundance in autophagy mutants, we circumvent some of these problems to reveal subsets of proteins that are directly influenced by autophagic processes, with or without compensatory changes in protein synthesis. Similar approaches in human fibroblasts (Zhang et al., 2016) and *Drosophila melanogaster* (Vincow et al., 2019) have also pinpointed specific protein complexes and organelles that are differentially affected by autophagy in other organisms.

### Common changes in cytosolic protein abundance and degradation rates in *atg11* and *atg5*

The *atg11* and *atg5* lines used in this report were previously shown to be *bona fide* single gene mutants in Arabidopsis, to have impaired autophagic fluxes detected by vacuolar delivery of *ATG8-GFP*, and to share typical early senescence phenotypes at late developmental stage and under nutrient limitation conditions (Thompson et al., 2005; Yoshimoto et al., 2009b; Li et al., 2014; Li and Vierstra, 2014). We show *atg11* and *atg5* share many common differences in cytosolic and organellar protein abundances and associated changes in protein degradation rates, although *atg5* typically showed larger relative changes in protein abundance (Figure 1;  Supplemental Figures S7 and S8). The larger differences in *atg5* are broadly consistent with the severity of this mutant’s senescence phenotype compared with *atg11* (Yoshimoto et al., 2009b; Li et al., 2014), and the established roles of ATG5 and ATG11 in the autophagy process; namely ATG5 acting in the core conjugation cascade and ATG11 acting in a regulatory complex.

Glycolytic enzymes including phosphoglycerate kinase (PGK), enolase, triosephosphate isomerase, and FBA showed slower protein degradation rates in both *atg11* and *atg5*, but these led to only a mild increase in abundance of these enzymes (0%–24%) in autophagy mutants (Figure 5;  Supplemental Data Set 6). Interactions between autophagy and glycolytic enzymes have been previously reported in plants and other organisms and can be complex (Han et al., 2015; Henry et al., 2015; Watson et al., 2015; Qian et al., 2017a, 2017b). Firs, glycolytic enzymes play roles in autophagic flux regulation. For example, glyceraldehyde-3-phosphate dehydrogenases (GAPDHs) negatively regulate autophagy in Arabidopsis (Henry et al., 2015) and in *Nicotiana benthamiana* GAPDHs can reduce autophagy activities by binding to ATG3 (Han et al., 2015). In contrast, PGK1 can induce autophagy under cellular stress conditions in mammals through phosphorylating of Beclin1 (Qian et al., 2017b). Second, autophagy can downregulate glycolysis metabolism through selective degradation of enzymes. For example, hexokinase is selectively degraded in human liver cancer cells during autophagy (Jiao et al., 2018). Here we show the basal rate of degradation of glycolytic enzymes in Arabidopsis is partially due to autophagy, but impaired autophagy may be compensated for by changes in glycolytic enzyme synthesis that prevent their accumulation. To provide independent confirmation we showed through protein localization and inhibitor studies that FBA8 can be degraded by autophagy in plants (Figure 6;  Supplemental Figure S12). Recently, proximity-dependent biotinylation screening of in vivo interactions confirmed GAPDH and FBA are bound by ATG8 in plants (Macharia et al., 2019). Together this evidence suggests autophagy plays a role in glycolytic enzymes maintenance, potentially through selective degradation.

The CCT protein complex in human cell lines is present in immunopurified autophagosomes, degrades slowly in autophagy mutants (Dengjel et al., 2012; Zhang et al., 2016), and can restrict neuropathogenic protein aggregation via autophagy in human cell lines and fruit fly (Pavel et al., 2016). Although well documented in animals, a 20S protein complex consisting of eight CCT subunits was only recently reported in plants (Ahn et al., 2019; McWhite et al., 2020). In this study, we established that five CCT protein complex subunits (CCT1–4 and 8) increased in abundance and had slower rates of degradation in roots of both autophagy mutants (Figure 5;  Supplemental Figure S7; Supplemental Data 6), supporting the hypothesis that CCT is an autophagy target in Arabidopsis roots. CCT is known to affect the folding and stability of tubulin in Arabidopsis and mutants with deficient CCT function show depletion of cortical microtubules and reduced alpha and beta tubulin abundance due to increased degradation (Ahn et al., 2019). Interestingly, we found that five beta tubulins (TUB2, 4, 6, 8, and 9) but not alpha tubulins, showed decreased abundance in *atg11* and *atg5*. It is unclear whether the decreased abundance of beta tubulin is a direct or indirect effect of impaired autophagy, but microtubules are important for autophagy. Microtubules can interact with autophagic proteins and play roles in preautophagosome structure, autophagy induction, formation, and movement (Mackeh et al., 2013). In plants, microtubules are proposed to aid autophagosome delivery to the vacuole with the help of FYVE and coiled-coil domain-containing proteins that bind ATG8 and PI3K on the autophagosome outer membrane (Marshall and Vierstra, 2018). We also found actin (ACT7) and the actin-interacting proteins VILIN4 and PROFILIN1/2 showed decreased abundance in both mutants. In yeast, actin filaments are only involved in selective but not bulk autophagy (Hamasaki et al., 2005; Reggiori et al., 2005; Monastyrska et al., 2009). In mammals, actin was found to be required for both selective and bulk autophagic degradation (Kast and Dominguez, 2017; Xu et al., 2018). In plants, actin filaments seem to be dispensable for bulk autophagy in *N.*  *benthamiana* (Zheng et al., 2019), but it is unclear whether actin is needed for any form of selective autophagy in plants. Our results thus suggest that the homeostasis of the plant CCT complex is controlled by autophagic degradation and that accumulation of CCT complex subunits correlated with decreases in the abundance of components of the cytoskeleton, which warrants further investigation.

### Changes in organelle abundance and degradation rates in *atg11* and *atg5*

Many proteins with decreased degradation rates in both root and shoot tissues of autophagy mutants localize to the ER, peroxisomes, or mitochondria (Figure 3). The number of proteins found to meet statistical thresholds in each organelle is still small using our approach, so we stop short of claiming whether these data represent selective autophagy of specific proteins or general changes in engulfment of whole organelles or a combination. However, the data clearly support the notion that a range of ER, peroxisome and mitochondrial proteins, if not whole organelles, are autophagy cargo in both photosynthetic and nonphotosynthetic tissues. Interestingly, we found that in roots of *atg5* and *atg11* plants, a high proportion of these proteins increased in abundance, while in shoots a high proportion decreased in abundance (Figure 1). This means that proteins with slower degradation showed reduced abundance in shoot but accumulated in roots, suggesting that photosynthetic tissues have more plasticity for transcription and translational control during autophagy than root tissues. The more prominent deployment of alternative proteostasis/protein recycling mechanisms in shoots than in roots is also consistent with the more drastic decrease in amino acids levels in roots than in shoots of autophagy mutants (Figure 7).

In roots, organelle proteins with faster degradation rates in autophagy mutants were predominantly localized to plastids, apoplast, plasma membrane, and vacuoles. Whereas we did not find evidence of increases in plastid protease abundance in roots (Supplemental Data Set 1), other degradative pathways can deliver portions of plastids to the vacuole (Izumi et al., 2017; Otegui, 2018; Zhuang and Jiang, 2019). Therefore, it is possible that ATG5/ATG11-independent pathways that mediate plastid turnover are stimulated in roots of autophagy-deficient mutants. The content of extracellular, plasma membrane, and vacuolar proteins is closely associated with the rate of intracellular vesicle trafficking. Proteins reach the vacuole through the secretory and endocytic/endosomal pathways as well as through autophagy (Marty, 1999; Pereira et al., 2014; Zhang et al., 2014; Shimada et al., 2018). Deficient autophagy in *atg5* and *atg11* correlated with a decreased abundance of both tonoplast and vacuolar lumen proteins (Supplemental Figures S7 and S8). The outer membrane of the autophagosome is integrated into the tonoplast upon fusion and it is therefore assumed to supply large quantities of membrane to vacuoles. Although not statistically significant, we noticed by electron microscopy a consistent decrease in tonoplast membrane in actively vacuolating cells of both *atg5* and *atg11* root cells. We also found that CLATHRIN HEAVY CHAIN 1 (At3g11130) was more abundant in roots of *atg5* (Supplemental Figure S7). Clathrin is associated with endocytosis at the plasma membrane and sorting at the TGN and endosomes (Gao et al., 2019). The altered abundance of clathrin and other trafficking components could alter both endocytosis/endosomal and exocytosis rates, contributing to the fast turnover and low abundance for both plasma membrane and extracellular proteins seen here in autophagy mutants. The endocytosis/exocytosis processes in *atg5* and other autophagy mutants merit further investigation.

### Protein abundance and degradation rate specific changes in *atg11* and *atg5*

The most severe molecular alterations in *atg5* were the five-fold increases in specific ER-resident proteins. This differential effect on ER homeostasis in *atg5* also correlated with accumulation of vesicle transport-associated proteins, such as three COP1 coatomers (alpha, delta, and gamma subunits) in *atg5*. COP1 is essential for retrieval of proteins with di-lysine motifs from Golgi stacks back to the ER (Wang et al., 2018), intra Golgi transport, and Golgi maintenance. Interestingly, we observed in *atg5* but not in *atg11* root cells’ abnormal membranous structures with assembled coats of unknown nature reminiscent of Golgi and/or ER membranes (Supplemental Figure S9). Whereas the origin of these abnormal, coated, membranous structures in *atg5* cells is unknown, the mis-regulation of COP1 components in mammalian cells induces the re-localization of Golgi, TGN, and ER markers into large membranous structures (Styers et al., 2008). The fungal toxin brefeldin A inhibits the assembly of the COP1 coat and also results in large abnormal membranous bodies (BFA bodies) in plants, that contain Golgi, TGN, and endosomal proteins (Nebenfuhr et al., 2002; Lam et al., 2009; Berson et al., 2014). In addition, the loss of COP1 subunits also leads to the accumulation of abnormal autophagosomes not fully capable to fusing with lysosomes (Razi et al., 2009).

A higher proportion of mitochondrial proteins with slower degradation rates was found in *atg11* compared with *atg5*, in both root and shoot tissues (Figure 4;  Supplemental Data Set 6). Higher abundance of mitochondrial proteins was also common in both shoots and roots of *atg11*, but only in shoots of *atg5* (Figure 1). ATG11 has been reported to be essential for senescence-induced mitophagy in Arabidopsis photosynthetic tissues (Li et al., 2014; Li and Vierstra, 2014) and ATG5-dependent mitophagy has been recently reported in Arabidopsis cotyledons and roots (Ma et al., 2021); however, in vivo changes in degradation rate of specific mitochondrial proteins in either *atg11* or *atg5* under control conditions have not been reported previously to our knowledge. Interestingly, although chloroplast proteins show general increases in abundance in shoots of both *atg11* and *atg5*, we only found a higher proportion of chloroplast proteins with slower degradation rates in shoots of *atg11* (Figures 2 and 4). These same chloroplast proteins showed faster protein turnover rates in *atg5*. We interpret this to mean that chloroplast proteins accumulated in *atg11* through deficient degradation but through enhanced synthesis in *atg5*. Taken together, the different patterns of degradation and abundance changes in mitochondrial and chloroplastic proteins in the mutant lines support a specialized role of ATG11 in basal level mitochondrial and chloroplast protein homeostasis and highlight specific organelle proteins that are good indicators of this role.

### Pi limitation caused autophagy-dependent cytosolic protein degradation and vacuole biogenesis in roots, and chloroplast degradation in shoots

Pi limitation has been reported to induce autophagy in yeast and plants (Tasaki et al., 2014; Yokota et al., 2017; Naumann et al., 2019) and we could reproduce this effect in hydroponically grown Arabidopsis plants (Supplemental Figure S13). Pi limitation-induced autophagy is reported to contribute to vacuole biogenesis (Gao et al., 2017), which is consistent with the general increase in abundance of vacuolar proteins in roots of *Wt* plants grown in Pi-limiting conditions (Supplemental Figure S15). A similar increase in vacuolar proteins was observed in roots of *atg11* but not of *atg5*, suggesting ATG11 is not essential for autophagy-dependent vacuole biogenesis under Pi-limiting conditions. A new ATG1-independent autophagy mechanism in prolonged carbon starvation conditions was recently reported (Huang et al., 2019), so this might explain the activation of an autophagic pathway independent of the ATG1 kinase complex in *atg11*. However, the role of ATG11 in mitochondrial degradation was not diminished under Pi limitation (Figure 8C), indicating that ATG1-independent autophagy in *atg11* cannot compensate for deficient mitochondria degradation. We also found that Pi limitation attenuated the cytosolic protein abundance differences observed between *Wt* and autophagy mutants, without affecting protein degradation rates. This finding could indicate complementary transcription/translation changes induced by Pi limitation in these autophagy mutants.

In roots, BGLU22 and ATGSTF8 had faster turnover rates under Pi limitation in *Wt* but not in either autophagy mutant (Figure 8G). While BGLU22 showed a slight abundance reduction, ATGSTF8 accumulated in *Wt*. This finding indicates that in *Wt* plants growing in Pi-limiting conditions, BGLU22 turnover is associated with the stimulated autophagic degradation while ATGSTF8 increased turnover is compensated with even greater protein synthesis. BGLU22 localizes to root ER bodies and is an EE-type myrosinase that can break down aliphatic glucosinolates during stress conditions (Sugiyama and Hirai, 2019). The faster degradation of BGLU22 under Pi-starvation suggest that ER bodies containing BGLU22 may be delivered to the vacuoles containing the aliphatic glucosinolates by autophagy.

All six proteins that degraded faster under Pi limiting conditions in *Wt* shoots were chloroplast-resident proteins. Four of them showed unchanged or slower degradation rate in *atg11* and *atg5* (Figure 9G), suggesting a role for Pi limitation in ATG11/5 associated autophagic chloroplast degradation. These unchanged or slower degrading proteins were components of photosystems and their light harvesting complexes (PSBO-1, PSAC, and LHCA3), and a plastid thioredoxin (thioredoxin M1) that regulates photosynthetic acclimation in fluctuating light intensities by regulating the export of excess reductive power from the chloroplasts (Thormahlen et al., 2017). An electron carrier between photosystems (plastocyanin 1) showed slower degradation and a significant increase in abundance in *atg11* but faster degradation rate in *atg5* and no change in abundance, which suggest its degradation is dependent on ATG11 but not ATG5 (Supplemental Data Set 10). These represent useful protein markers to study general or selective chloroplast degradation by autophagy.

## Materials and methods

### Arabidopsis hydroponic plants preparation and ^15^N labeling

*Arabidopsis thaliana* accession Columbia-0 (Col-0; *Wt*), *atg5* and *atg11* plants were grown under 16/8-h light/dark conditions with cool white T8 tubular fluorescent lamps 4000 K 3350 lm (Osram, Munich, Germany) with intensity of 100–125 μmol m^−2^ s^−1^ at 22°C. The hydroponic protocol was as described previously (Waters et al., 2012) and used a modified Hoagland solution (2-mM CaCl_2_, 6-mM KNO_3_, 0.5-mM NH_4_NO_3_, 0.5-mM MgSO_4_, 0.25-mM KH_2_PO_4_, 0.05-mM KCl, and 0.04-mM Fe-EDTA) supplemented with micro elements (25--μM H_3_BO_3_, 2-μM MnCl_2_, 2-μM ZnSO_4_, 0.5-μM CuSO_4_, 0.15-μM CoCl_2_ and 0.25-μM (NH_4_)_6_Mo_7_O_24_) and 2.6 mM MES, and the pH was adjusted to 5.8–6.0. Seeds of different lines (*Wt*, *atg5*, and *atg11*) were planted on the growth hole of agar stuffed in lids of 1.5-mL black tubes sitting in 24-well floater tube racks containing 160 mL growth medium. The seeds were stratified under 4°C for 2–3 days before being transferred to the growth chambers. Half-strength growth medium was used for the first week. A single plant was placed in every tube lid and four tubes lids in each floater tube rack (Supplemental Figure S1). The growth medium was changed every 5 days. Arabidopsis plants were grown for 21 days in natural abundance medium until they reached leaf production stage 1.10 (T0; Boyes et al., 2001) as noted. Arabidopsis plants of this age reach a steady-state proteome, allowing single time point protein turnover measurements to be equivalent to multi time point measurement as describe previously (Li et al., 2017). Mass spectra of peptides in single time points after label addition by this method show the cumulative synthesis and degradation of natural abundance and labeled peptide populations. To obtain a fully labeled ^15^N protein reference standard, ^15^N medium (with 6-mM K^15^NO_3_, 0.5-mM ^15^${\text{NH}}_{4}^{15}$NO_3_) was used to replace the natural abundance nitrogen in the medium and plants were grown from seed in this medium for 26 days. For progressive ^15^N labeling, the growth medium was discarded and the growth racks rinsed 4 times with fresh medium without nitrogen (no KNO_3_ or NH_4_NO_3_) to ensure the old solution was washed out. A total of 160 mL of ^15^N medium (6-mM K^15^NO_3_, 0.5-mM ^15^${\text{NH}}_{4}^{15}$NO_3_) was added for every four plants and the plants were grown for 3 days before collecting leaf and root tissues for separate total protein extraction (Supplemental Figure S17). Root/shoot from two plants in one rack were pooled as a biological replicate, and three biological replicates were collected.

### Protein extraction, in-solution digestion, high pH high-performance liquid chromatography (HPLC) separation, and LC–MS analysis of tryptic peptides

The root/shoot samples (0.1–0.2 g) from fully ^15^N labeled reference, 15N progressively labeled and unlabeled of three lines (*Wt*, *atg5*, and *atg11*) were snap frozen in liquid nitrogen and homogenized using Qiagen tissue lysis beads (5 mm) by vortex. A total plant protein extraction kit (PE0230-1KT, Sigma Chemicals) was used to extract root/shoot total proteins. The final pellet of total protein was dissolved in Solution 4 and then reduced and alkylated by tributylphosphine and iodoacetamide as described in the Sigma manual. The suspension was centrifuged at 16,000 *g* for 30 min at 4°C and the supernatant was assay for protein concentration by amido black quantification as described previously (Liu et al., 2012a).

A total of 100 µg root/shoot proteins from progressively ^15^N labeled samples were digested in solution as described previously (Nelson et al., 2014). A total of 50 µg of unlabeled root/leaf protein samples noted above was mixed individually with 50 µg of the fully ^15^N-labeled reference and digested in solution by trypsin. Each sample was separated into 96 fractions by high pH HPLC separation and further pooled into 12 fractions and each fraction was analyzed by MS. Filtered samples (5 µL each) were loaded onto a C18 high-capacity nano LC chip (Agilent Technologies, Santa Clara, CA, USA) using a 1200 series capillary pump (Agilent Technologies) and the following buffer B (0.1% FA in Acetonitrile) gradient: 5%–35% in 35 min, 35%–95% in 2 min and 95%–5% in 1 min. Peptides were eluted from the C18 chip directly into a 6550 series quadrupole time-of-flight (Q-TOF) mass spectrometer (Agilent Technologies) with parameter settings described previously (Li et al., 2017).

### MS data analysis, calculations of *K*_D_, and relative abundance values

Agilent .d files were converted to mzML using the Msconvert package (version 2.2.2973) from the Proteowizard project, and mzML files were subsequently converted to Mascot generic files using the mzxml2 search tool from the TPPL version 4.6.2. Mascot generic file peak lists were searched against an in-house Arabidopsis database comprising ATH1.pep (release 10) from The Arabidopsis Information Resource and the Arabidopsis mitochondrial and plastid protein sets (33,621 sequences; 13,487,170 residues; Lamesch et al., 2012), using the Mascot search engine version 2.3 and utilizing error tolerances of 100 ppm for MS and 0.5 Da for MS/MS; “Max Missed Cleavages” set to 1; variable modifications of oxidation (Met) and carbamidomethyl (Cys). We used iProphet and ProteinProphet from the Trans Proteomic Pipeline to analyze peptide and protein probability and global false discovery rate (FDR; Nesvizhskii et al., 2003; Deutsch et al., 2010; Shteynberg et al., 2011). The reported peptide lists with *P* = 0.8 have FDRs of <3% and protein lists with *P* = 0.95 have FDRs of <0.5%. Quantification of LPFs were accomplished by an in-house R script which was written originally in Mathematica (Nelson et al., 2014). A median polish method described previously was used for data analysis (Li et al., 2017). Measured protein degradation rate 0.1 d^−1^ was used to calculate the FC protein (FCP) for shoot samples. For root tissues, measured FCP based on fresh weight was measured before and after progressive ^15^N labeling. A measured degradation rate 0.5 day ^−1^ was determined and then applied to calculate FCP in samples of *Wt* and mutant lines, which were applied for degradation rate calculations. We determined changes in specific protein abundance using a fully labeled ^15^N protein reference standard. Protein abundance was represented as ratio to reference and normalized to all samples (three lines under both control and Pi starvation conditions) as previously reported (Li et al., 2017). Trypsin was used as protease and up to two missed cleavages were allowed in matching tryptic peptides to Arabidopsis sequences. The MS mzml files were processed in MaxQuant software (version 2.0.2.0) using default settings for LFQs. Cysteine carbamidomethylation was set up as a fixed modification and oxidation of methionine and N-terminal acetylation as a variable modification. Peptide identification required a minimum of seen peptides and a maximum of five modifications was allowed. Match-between-run function was enabled. Global data normalization was done using the MaxQuant LFQ algorithm with LFQ minimum ratio count set to 2 and fast LFQ enabled. LFQ protein abundance measurements showed higher protein number compared with the ^15^N spike-in analysis and were used as a validation of ^15^N spike-in data and for comparison with previous reports (Supplemental Figures S2, S4, S5, S6, and S14). Relative ΔAbundance (i.e. (mutant−*Wt*)/Average (mutant and *Wt*)) was used to describe the level of changes between mutant versus *Wt* or treatment versus control.

### RNA extraction and qPCR analysis

We collected the fifth true leaf from all three lines at 21 days, and collected leaf 6 after 3 days Pi starvation treatment. The shoot samples (∼0.1 g) from three lines (*Wt*, *atg5*, and *atg11*) under control/Pi starvation conditions were snap frozen in liquid nitrogen and homogenized to powder using Qiagen tissue lysis beads (2 mm) by a homogenizer. RNA was extracted using a Spectrum Plant Total RNA kit (Sigma-Aldrich, St. Louis, MO, USA; STRN250-1KT) with On-Column DNase treatment (Sigma-Aldrich; DNASE70) following manufacturer’s instructions. About 500 ng of RNA was used for cDNA synthesis with an iScript cDNA synthesis kit (Bio-Rad, Hercules, CA, USA; 1708890). Transcripts of *spx1 (F-TGCCGCCTCTACAGTTAAATGGC*, *R-TGGCTTCTTGCTCCAACAATGG*), *atg8h (F-TGCAGTTAGATCCATCCAAAGCTC*, *R-TCCATGCGACTAGCGGTTTGAG*), and *atg7 (F-ACGTGGTTGCACCTCAGGATTC*, *R-ACTAAGAGTTCAACGGCGAGAGC*) were quantified using a QuantiNova SYBR green PCR kit (Qiagen, Hilden, Germany; 208056) with LightCycler380 in *Wt*, *atg5/11* lines under both control and Pi starvation conditions. We did four biological replicates for most samples except *atg11* poststarvation treatment. QPCR data were normalized to housekeeping genes AKT2 *(F-GGTAACATTGTGCTCAGTGGTGG*, *R-AACGACCTTAATCTTCATGCTGC*) and UBQ10*(F-CTGCGACTCAGGGAATCTTCTA*, *R-TTGTGCCATTGAATTGAACCC*) and then analyzed using geometric averaging of multiple control genes (Vandesompele et al., 2002; Czechowski et al., 2005) before being compared.

### Protoplast transformation and confocal imaging

FBA8 (At3g52930) was amplified from Arabidopsis cDNA using primers containing *Sac I* and *Afl II* cutting sites (F-*AAGAGCTCATGTCTGCCTTCACAAGC*, R-*CGCCTTAAGGTACTTGTAATCCTTCAC*). DsRed was amplified from a host vector using primers that contain the Afl II and Not I cutting site (F-*GCGCTTAAGATGGCCTCCTCCGAGGACGTC*, R-*TTGCGGCCGCTTACAGGAACAGGTGGTGGCG*). A transient transformation vector *pHBT-sGFP(S65T)-NOS* was cut by Sac I and Not I before a ligation reaction of three fragments catalyzed by T4 ligase. The constructed *pHBT-sGFP(S65T)-NOS FBA8-DsRed* was sequenced and used for protoplast transient transformation. Transgenic Col-0 Arabidopsis plants expressing *ProUBQ10:GFP-ATG8a* (Kim et al., 2013) were used for generating protoplasts. Protoplasts were isolated as previously described (Wu et al., 2009). Approximately 12 μg of the *pHBT-sGFP(S65T)-NOS FBA8-DsRed* vector was used for each transformation. Protoplasts for confocal microscopy imaging were incubated at 23°C in darkness for 12–14 h before imaging. To visualize autophagosomes and autophagic bodies, protoplasts were cultured in the presence of 0.5 µM AZD8055 (dissolved in DMSO) with or without 0.5-µM ConA (dissolved in DMSO) before imaging. As controls, protoplasts were also treated individually with DMSO or 0.5-µM ConA.

All fluorescence microscopy images were captured on a 780 Zeiss laser scanning confocal microscope. Arabidopsis leaf protoplasts were loaded onto an 18 Well Flat µ-Slide (Ibidi, Cat. No. 81826) and imaged using a 63× water immersion objective (numerical aperture 1.46). The multitrack mode was used for sequentially imaging of GFP and DsRed, and chlorophyll. GFP was excited with a -nm laser line and detected using a 493–546 nm band-pass filter, DsRed was excited with a 561-nm laser line and detected using a 570–615 nm band-pass filter, and chlorophyll was excited with a 633-nm laser line and detected using a 647–721 nm band-pass filter. The fluorescence profiles of GFP and DsRed were measured using the “Profile” tool of ZEN Lite version 3.2.

### Immunoblot-linked protein degradation assay

Seven-day-old plate grown Arabidopsis seedlings were treated in half-strength Hoagland media supplied with DMSO, 20-μM E64d and 1-μM ConA for 12 h before being harvested and store at −80°C. Seedlings were homogenized in liquid nitrogen to a fine powder before addition of total protein extraction buffer (50-mM Tris pH 7.5, 150-mM NaCl, 0.1% sodium dodecyl sulfate (SDS), 1% Triton X-100, 0.5% Na-deoxycholate, 1-mM EGTA, 1-mM DTT, 1× complete protease inhibitor cocktail, 1-mM PMSF). Protein samples in extraction buffer were centrifuged at 20,000 *g* for 30 min at 4°C to precipitate insoluble material. The concentration of total protein in the supernatant was determined using Amidoblack before proteins were separated by SDS–PAGE. Proteins were blotted to a PVDF membrane and incubated in a rabbit polyclonal antibody against FBA8 (PhytoAB, PHY2192S with 1:4,000 dilution). A goat anti-rabbit antibody (Agrisera, Vännäs, Sweden; AS09602 with 1:10,000 dilution) was added for visualization.

### Pi concentration measurement by a colorimetric assay

*Wt*, *atg5*, and *atg11* lines were grown hydroponically till leaf production stage 1.10 (T0). Growth containers were rinsed with water for complete phosphate depletion. For Pi starvation treatment, plant growth media was replaced with Hoagland solution without phosphate and grown for 3 days (T3-Pi starvation). Hoagland solution with phosphate was used for control plants (T3). Inorganic concentration in root/shoot tissues of three lines were measured by a colorimetric assay. Inorganic phosphate was extracted in 500 µL water from 10-mg frozen powdered samples. The concentration of Pi was determined spectrophotometrically at 820 nm after a 90-min reaction at 37°C in the presence of 1.4% w/v ascorbate and 0.36% w/v ammonium molybdate in 1 N H_2_SO_4_ (Ames, 1966).

### Maximum quantum yield of PSII measurement by IMAGING-PAM

Leaf production stage 1.10 Arabidopsis plants (T0-grown in hydroponics for ∼21 days postgermination) were washed by Hoagland media without phosphate and then grown for another 3 days in fresh growth media (T3-control) or growth media without phosphate (T3-phosphate starvation). Whole plants were dark adapted for at least 20 min before being measured by a MAXI version of the IMAGING-PAM. A color gradient was used to demonstrate the Fv/Fm (maximum quantum yield of PSII) values, which were measured by IMAGING-PAM in leaves of the whole rosette. One biological replicate was a combination of mean measured Fv/Fm values in two Arabidopsis plants.

### Autophagic flux assay by confocal laser scanning microscopy

GFP-ATG8a plants were grown hydroponically till leaf production stage 1.10. Whole plants were transferred into normal or Hoagland media without phosphate and grown for another 3 days. E64d or concanamycin A was supplemented into growth media 24 h before the confocal laser scanning microscopy experiment to a final concentration of 100-µM E64d and 1-µM ConA. A Nikon A1Si confocal microscope equipped with laser line 488-nm excitation and emission band-pass filter of 500–520 nm, and controlled by a NIS element AR software package (version 4.13.01, Build 916) was used. Images were acquired using a 20× lens (Nikon CFI Plan Apo VC 20× 0.75 N.A.) with pinhole diameter of 2.5 airy units (corresponds to the optical slice of 4.37 um). Autophagic puncta (AP) of representative images were counted by the “Analyze Particles” function of ImageJ. AP numbers in each Z-stack were plotted. The distribution of AP number under control and Pi limitation conditions in the representative image were compared by Kolmogorov–Smirnov test for significance.

### Transmission electron microscopy

Arabidopsis seedlings were grown for 24 days in hydroponic conditions as described above. Root tips were excised and placed in freezing planchettes containing 0.1-M sucrose and high-pressure frozen in a Baltec HPM 010. Samples were high-pressure frozen in 2% (w/v) OsO_4_ in anhydrous acetone in dry ice overnight and warmed to room temperature on a rocker with slow agitation for several hours, until they reached room temperature. After several acetone rinses and the planchettes removed, samples were infiltrated in a series of Epon resin changes polymerizing at 60°C for 24 h. Sections were stained with 2% uranyl acetate and lead citrate (2.6% lead nitrate and 3.5% sodium citrate, pH 12) and imaged in a Philips CM120 transmission electron microscope. Morphological measurements were done using FIJI (Schindelin et al., 2012).

### Metabolite extraction

Plant tissues (15–50 mg) were collected at specified time points and immediately snap-frozen in liquid nitrogen. Samples were ground to fine powder and 500 μL of cold metabolite extraction solution (90% [v/v] methanol, spiked with 2 mg/mL ribitol, 6 mg/mL adipic acid, and 2 mg/mL and ^13^C-leucine as internal standards). Samples were immediately vortexed and shaken at 1,400 rpm for 20 min at 75°C. Cell debris was removed by centrifugation at 20,000*g* for 5 min at 4°C. For each sample, 100 or 400 μL of supernatant was transferred to a new tube and either proceeded to derivatization for liquid chromatography mass spectrometry (LC–MS) analysis or dried using a SpeedVac.

### Analyses of SA, organic acids, and amino acids by selective reaction monitoring using QQQ-MS

For LC–MS analysis of organic acids, sample derivatization was carried out based on previously published methods with modifications (Han et al., 2013). Briefly, for each 100 µL of sample, 50 µL of 250-mM 3-nitrophenylhydrazine in 50% methanol, 50 µL of 150-mM 1-ethyl-3-(3-dimethylaminopropyl) carbodiimide in methanol, and 50 µL of 7.5% pyridine in 75% methanol were mixed and allowed to react on ice for 60 min. To terminate the reaction, 50 µL of 2 mg mL^–1^ butylated-hydroxytoluene in methanol was added, followed by the addition of 700 µL of water. Derivatized organic acids were separated on a Phenomenex Kinetex XB-C18 column (50× 2.1 mm, 5-µm particle size) using 0.1% formic acid in water (solvent A) and methanol with 0.1% formic acid (solvent B) as the mobile phase. The elution gradient was 18% B at 1 min, 90% B at 10 min, 100% B at 11 min, 100% B at 12 min, 18% B at 13 min, and 18% B at 20 min. The column flow rate was 0.3 mL/min and the column temperature was maintained at 40°C. The triple quadrupole (QQQ)-MS was operated in the negative ion mode with multiple reaction monitoring (MRM) mode.

For measuring SA and amino acids, dried samples were resuspended in 100-µL HPLC-grade water before they were filtered to remove insoluble debris. Metabolites were separated on an Agilent Poroshell 120 Bonus-BP column (100 × 2.1 mm, 2.7-µm internal diameter) using 0.1% formic acid in water (solvent A) and acetonitrile with 0.1% formic acid (solvent B) as the mobile phase. For the analysis of amino acids and sugars, the elution gradient was 0% B at 1 min, 1% B at 4 min, 10% B at 6 min, 100% B at 6.5 min, 100% B at 8 min, 0% B at 8.5 min, and 0% B at 15 min. The column flow rate was 0.25 mL min^–1^, the column temperature was kept at 40°C. The QQQ-MS was operated in the positive ion mode with MRM mode. For SA, the elution gradient was 0% B at 1 min, 1% B at 3 min, 95% B at 23 min, 100% B at 23.2 min, 100% B at 25 min, 0% B at 25.5 min, and 0% B at 34 min. The column flow rate was 0.20 mL min^–1^ and the column temperature was set to 40°C. The LC–MS was operated in the negative ion mode with MRM mode.

A 0.5-µL or a 15-µL aliquot of each sample was injected and analyzed by an Agilent 1100 HPLC system coupled to an Agilent 6430 QQQ mass spectrometer equipped with an electrospray ion source. Data acquisition and LC–MS control were done using the Agilent MassHunter Data Acquisition software (version B06.00 Build 6.0.6025.4). The autosampler was kept at 10°C. The QQQ-MS was operated in MRM mode using the following operation settings: capillary voltage, 4000 V; drying N_2_ gas and temperature, 11 L min^–1^ and 125°C, respectively; Nebulizer, 15 psi. All optimized MRM transitions for each target were listed in Supplemental Data Set 12. All data were analyzed using MassHunter Quantitative Analysis Software (version B.07.01, Build 7.1.524.0). Metabolites were quantified by comparing the integrated peak area with a calibration curve obtained using authentic standards, and normalized against fresh weight and internal standards.

### Measurement and identification of sugars and secondary metabolites by Q-TOF MS

Analyses of sugars and secondary metabolites were performed using an Agilent 1100 HPLC system coupled to an Agilent 6510 Q-TOF mass spectrometer equipped with an electrospray ion source. Data acquisition and LC–MS control were carried out using the Agilent MassHunter Data Acquisition software (version B02.00). Separation of metabolites was performed using a Luna C18 column (Phenomenex; 150 × 2 mm, 3-µm particle size). The mobile phase consisted of 97:3 water:methanol with 10-mM tributylamine and 15-mM acetic acid (solvent A) and 100% methanol (solvent B). The gradient program was 0% B 0 min, 1% B 5 min, 5% B 15 min, 10% B 22 min, 15% B 23 min, 24% B 25 min, 29% B 80 min, 95% B 81 min, 95% B 82 min, 0% B 83 min, and 0% B 97 min. The flow rate was 0.2 mL/min, with column temperature kept at 35°C and samples at 10°C. The Q-TOF was operated in MS mode with negative ion polarity using the following operation settings: capillary voltage, 4000 V; drying N_2_ gas and temperature, 10 L min^–1^ and 250°C, respectively; Nebulizer, 30 psi. Fragmentor, skimmer, and octopole radio frequency (Oct1 RF Vpp) voltages were set to 110, 65, and 750 V, respectively. The scan range was 70–1,200 *m/z* and spectra were collected at 4.4 spectra/s, which corresponded to 2,148 transients/spectrum. All MS scan data were analyzed using MassHunter Quantitative Analysis Software (version B.07.01, Build 7.1.524.0). Peaks were normalized against sample weight and the internal standard. For identification of metabolites without authentic standards, Q-TOF was operated in Targeted MS/MS mode with negative ion polarity using the same MS settings as outlined above. The MS/MS scan range was 40–1,000 *m/z* and spectra were collected at 3.7 spectra/s which corresponded to 2,603 transients/spectra. For each metabolite target, the retention time window was set to ±1 min, isolation width was set to narrow (∼1.3 *m/z*), 10- to 20-, and 40-eV collision energies were used and the acquisition time was set to 180 ms/spectra. The identity of each unknown was verified by comparing MS/MS fragment ions with published data (Stobiecki et al., 2006; Lee et al., 2008; Rochfort et al., 2008; Matsuda et al., 2009; Bartsch et al., 2010; Bialecki et al., 2010; Zhang et al., 2013; Lin et al., 2014; Hohner et al., 2018). The expected *m/z*, retention time and the method for identification were listed in Supplemental Data Set 12.

### Statistical analysis

Comparison and grouping of cellular compartments were acquired by *k*-samples distributions (Kruskal–Wallis) and multiple pairwise comparisons using the Conover–Iman procedure in XLSTAT software (Figure 1). One-way analysis of variance (ANOVA) followed by post-hoc Turkey test was performed for grouping of cellular compartments in transmission electron microscopy image analysis (Figure 2). Student’s *t* test (tails 2 and type 2) was performed in Excel to acquire *P*-values for comparisons of transcription, protein abundance, and turnover between *Wt* and mutant lines or between control and Pi limited conditions (Supplemental Data Sets 1, 3, 7, and 8; Supplemental Figure S10). Two-way ANOVA followed by Tukey’s ad-hoc was carried out using Sigmaplot software for metabolite analysis. Statistically significant changes with *P* ˂0.01 or 0.05 between *Wt* and mutant lines or between control and Pi limited conditions were reported (Figure 7). PCA was performed for genotypes and treatments at a 95% significance level in Multiple Experiment Viewer (Figures 8 and 9) and XLSTAT (Supplemental Figures S2 and S14) software. A Two-sample Kolmogorov–Smirno test was utilized for comparison of distributions of relative changes in protein abundance and degradation rate of cellular localizations in control and Pi limited conditions (Figures 8 and 9;  Supplemental Figures S15 and S16).

### Accession numbers

Gene accession numbers of *A.*  *thaliana* proteins reported in protein abundance and turnover datasets can be found in Supplemental Data Sets 1, 3, 7, and 8. MS data can be accessed through the Proteomexchange Consortium (https://www.proteomexchange.org/) through four entries: protein abundance measurements in root and shoot of *Wt*, *atg5*, and *atg11* under control and Pi limitation conditions (PXD010992 and PXD010948), protein turnover rates measurements in root and shoot of *Wt*, *atg5*, and *atg11* under control and Pi limitation conditions (PXD010900 and PXD010932).

## Supplemental data

The following materials are available in the online version of this article.

**Supplemental Figure S1.** Arabidopsis *atg5* and *atg11* phenotypes compared to *Wt* plants.

**Supplemental Figure S2.** A PCA of protein abundance in *atg5*, *atg11*, and *Wt* root and shoot.

**Supplemental Figure S3.** Changes in protein abundance by ^15^N spike-in in roots and shoots of Arabidopsis autophagy mutants.

**Supplemental Figure S4.** Changes in protein abundance by LFQ in roots and shoots of Arabidopsis autophagy mutants.

**Supplemental Figure S5.** Correlation analysis of protein abundance acquired from ^15^N spike-in and LFQ strategies.

**Supplemental Figure S6.** Significant changes in abundance of ribosome and proteasome subunits in Arabidopsis autophagy mutants.

**Supplemental Figure S7.** Significant changes in relative Δabundance of 241 root proteins in Arabidopsis autophagy mutants.

**Supplemental Figure S8.** Significant changes in relative Δabundance of 265 shoot proteins in Arabidopsis autophagy mutants.

**Supplemental Figure S9.** Abnormal organelles observed by microscopy in *atg5* root cells.

**Supplemental Figure S10.** Changes in protein degradation rate (K_D_) in roots and shoots of Arabidopsis autophagy mutants compared to *Wt*.

**Supplemental Figure S11.** Specific proteins that degrade more slowly in *atg5 and atg11* roots and shoots compared with *Wt* Arabidopsis.

**Supplemental Figure S12.** Arabidopsis glycolytic FBA8 accumulated in *atg5* and *atg11* mutant lines and its degradation was inhibited in E64d and ConA treated *Wt*.

**Supplemental Figure S13.** Pi limitation induces changes in *atg5*, *atg11*, and *Wt* Arabidopsis plants.

**Supplemental Figure S14.** An PCA to evaluate Pi limitation on LFQ in *atg5*, *atg11*, and *Wt*.

**Supplemental Figure S15,** Effects of Pi limitation on protein abundance in roots and shoots of *Wt* and autophagy mutants.

**Supplemental Figure S16.** Effects of Pi limitation on protein degradation rates in roots and shoots of *Wt* and autophagy mutants.

**Supplemental Figure S17.** Workflow of analysis to determine protein abundance, protein degradation rates, and metabolite abundances in samples from Arabidopsis plant tissues.

**Supplemental Data Set 1.** Changes in protein abundance in roots and shoots of hydroponically grown Arabidopsis autophagy mutants compared to *Wt*.

**Supplemental Data Set 2.** Changes in protein abundance of proteins belonging to different subcellular locations in autophagy mutants compared to *Wt*.

**Supplemental Data Set 3.** Changes in protein degradation rate in autophagy mutants.

**Supplemental Data Set 4.** Changes in protein abundance and degradation rate in autophagy mutants.

**Supplemental Data Set 5.** Proteins with significant differences in protein abundance and degradation rate.

**Supplemental Data Set 6.** Known and putative autophagy protein targets with significant changes in their abundance and degradation rate.

**Supplemental Data Set 7.** Changes in protein abundance in *Wt* and autophagy mutants under control and Pi limiting conditions.

**Supplemental Data Set 8.** Changes in protein degradation rate in *Wt* and autophagy mutants under control and Pi limiting conditions.

**Supplemental Data Set 9.** Protein degradation rates and protein abundance data for PCA.

**Supplemental Data Set 10.** Proteins with faster turnover rates under Pi limitation conditions in *Wt*.

**Supplemental Data Set 11.** Metabolites measurement using MS.

**Supplemental Data Set 12.** Precursor masses of metabolites used in LC–MS analysis.

## Supplementary Material

koac185_Supplementary_Data

## Notes

L.L. and A.H.M. designed the research. C.P.L., A.W.Y., and M.B. performed plant culture and biochemical experiments. X.D. and Y.Q. performed protoplast transformation and confocal microscopy, M.S.O. performed and analyzed TEM data. Mass spectrometry and analysis were performed by L.L. L.L., A.H.M., and M.S.O. contributing to the writing and revision of the article.

The authors responsible for distribution of materials integral to the findings presented in this article in accordance with the policy described in the Instructions for Authors (https://academic.oup.com/plcell) are Lei Li (lei.li@nankai.edu.cn) and A. Harvey Millar (harvey.millar@uwa.edu.au).
