# Supplementary material for: Defects in autophagy lead to selective in vivo changes in turnover of cytosolic and organelle proteins in Arabidopsis
Source: Plant Cell. 2022 Jun 29;34(10):3936–60. doi: 10.1093/plcell/koac185 (PMC9516138; doi:10.1093/plcell/koac185)
Supplement: koac185_Supplementary_Data [file koac185_supplementary_data.zip › koac185_Supplementary_Data/tpc.21.00373Supplemental.pdf]

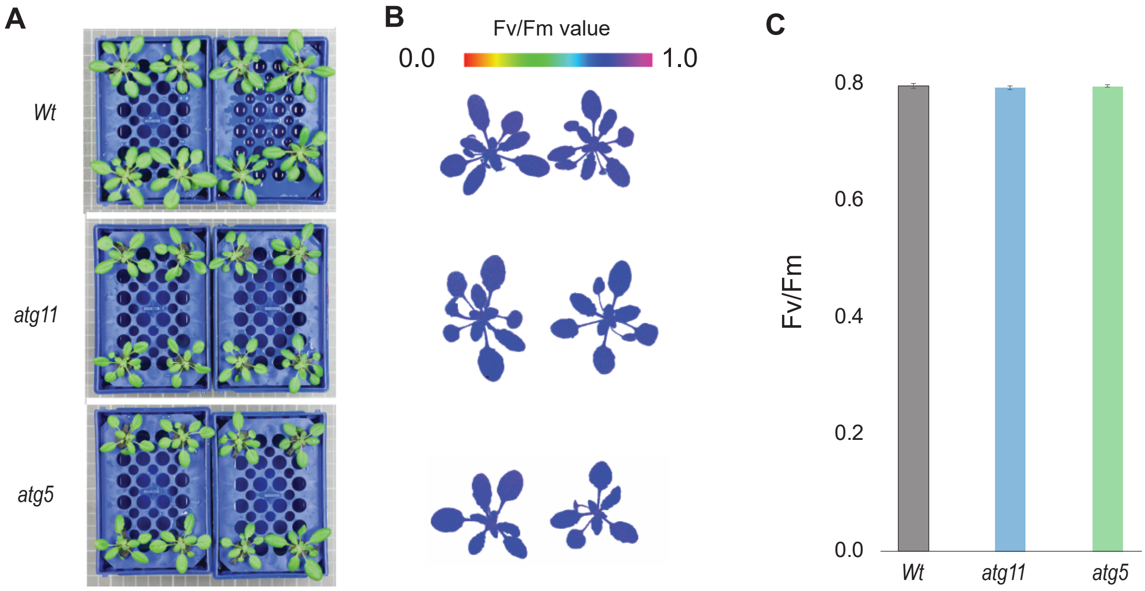

Supplemental Figure S1. Arabidopsis *atg5* and *atg11* phenotypes compared to *Wt* plants (Supports Figure 1.)

Arabidopsis plants (wild type (*Wt*), *atg5* and *atg11*) were grown in hydroponics for 21 days. Autophagy mutants showed the same phenotype as *Wt* control (**A**). A color gradient is used to show the maximum quantum yield of PSII (Fv/Fm) measured by IMAGING-PAM after 20 mins dark adaptation of the whole rosette. Fv/Fm values were evenly distributed in *Wt* and mutants leaves without any early senescence hot spots observed (**B**). Mean Fv/Fm values from four biological replicates were compared across Wt, *atg5* and *atg11* (**C**). Each biological replicate was a combination of Fv/Fm values from six leaves of two Arabidopsis plants.

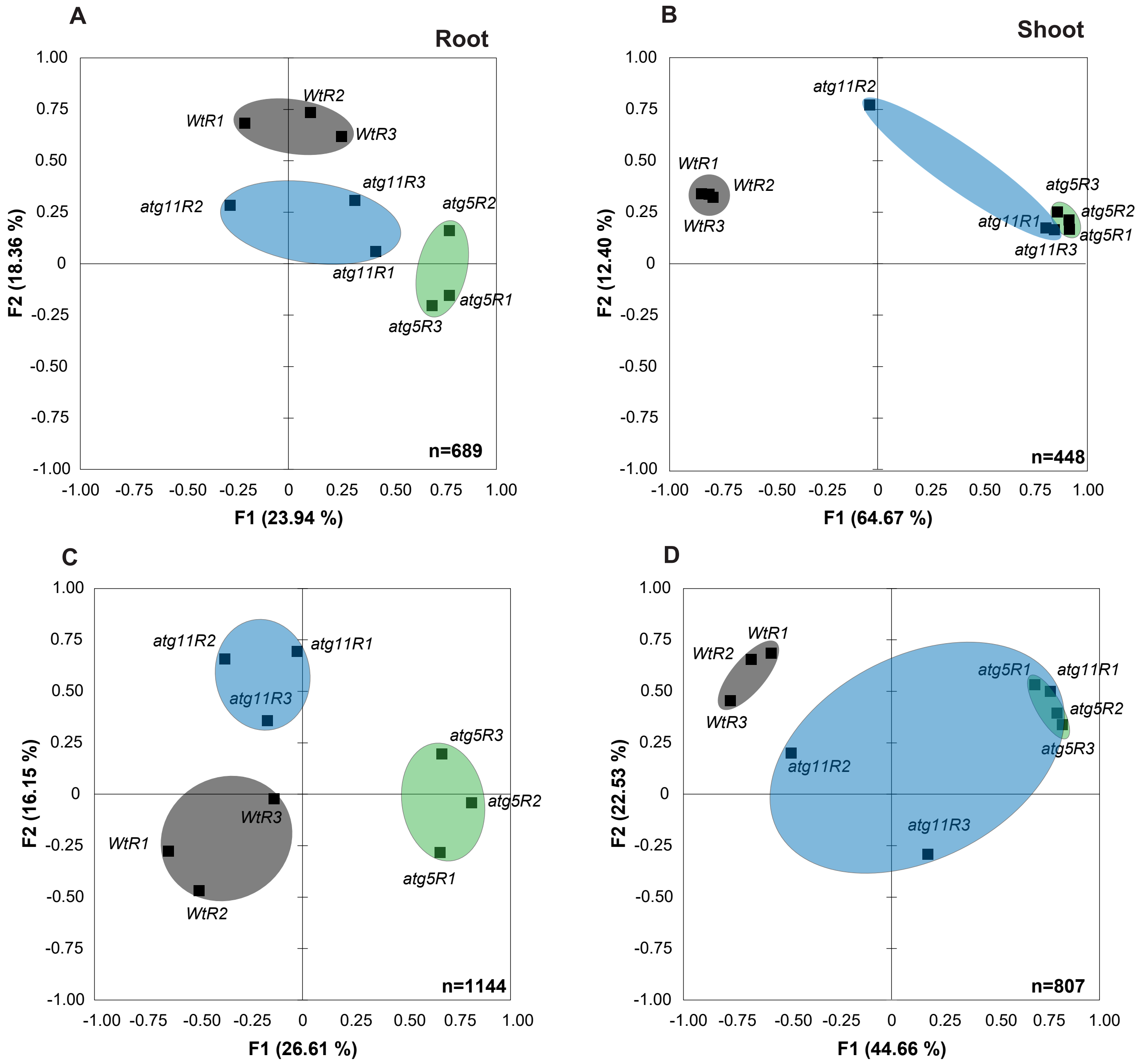

Supplemental Figure S2. A PCA of protein abundance in *atg5*, *atg11* and *Wt* root and shoot. (Supports Figure 1.)

Protein abundance measurements using label free ^{15}N spike-in (**A,B**) or label free quantification (**C,D**) were first normalized and used for principle component analysis (PCA). The number of proteins with measured abundance in all samples were shown in each graph. The full list of all proteins identified and quantified is shown in **Data S1**.

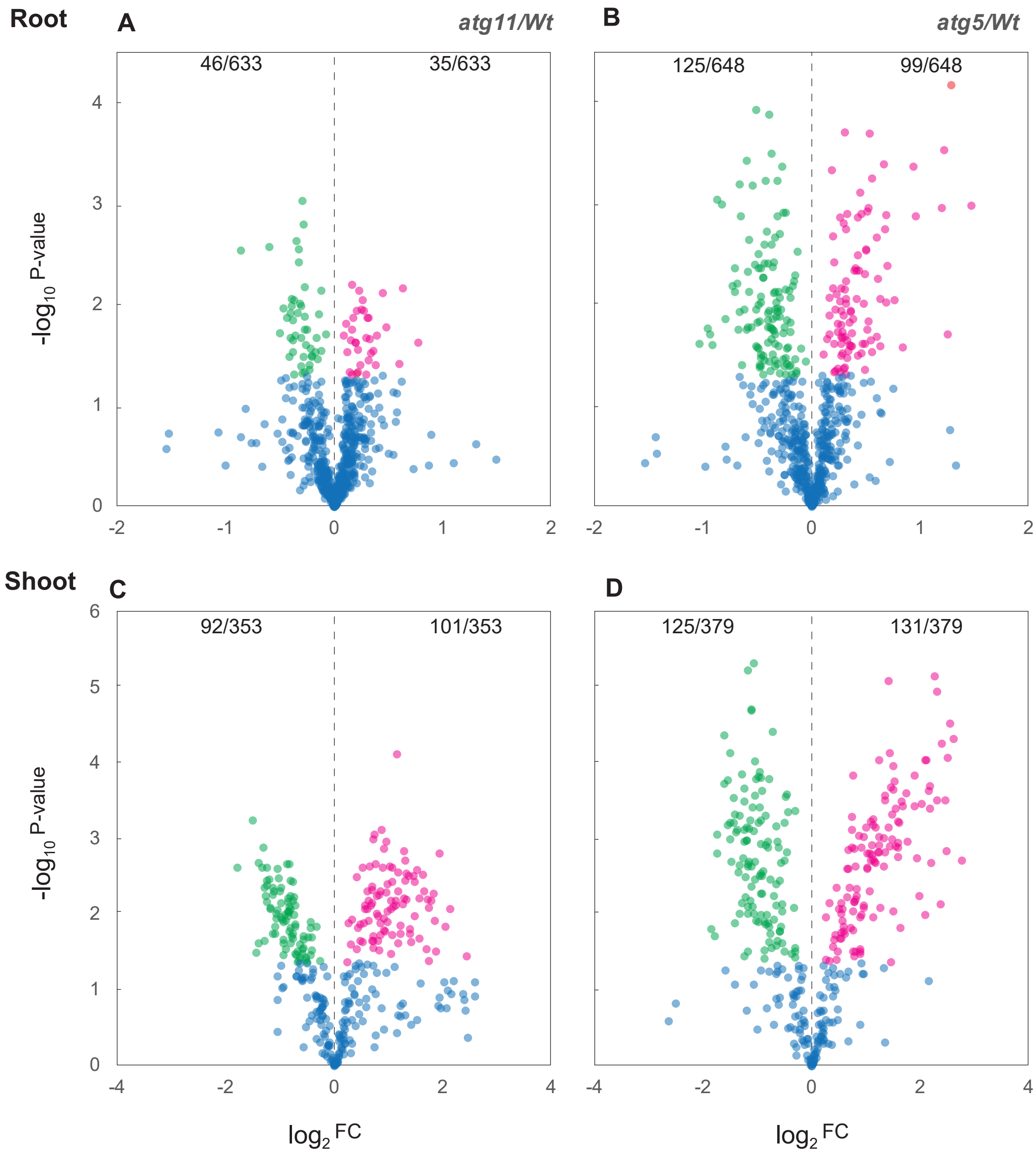

Supplemental Figure S3. Changes in protein abundance by ^{15}N spike-in in roots and shoots of Arabi-dopsis autophagy mutants.

(Supports Figure 1.)

Volcano plots of log2 fold change (FC) in protein abundance values between mutants and wild type roots (**A,B**) and shoot (**C,D**). Proteins with significant changes in abundance (P-value<0.05, Student's T-test) were colored magenta (up) or green (down) for visualization. In *atg11*, 13% of root proteins show significant changes in abundance (6% up, 7 % down) (**A**) while 55% of shoot proteins show significant changes in abundance (29% up, 26% down) in comparison with wild type (**C**). In *atg5*, 35% of root proteins show significant changes in abundance (15% up, 19% down) (**B**) while 68% of shoot proteins show significant changes in abundance (35% up, 33% down) in comparison with wild type (**D**). The full list of all proteins identified and quantified are shown in **Data S1**.

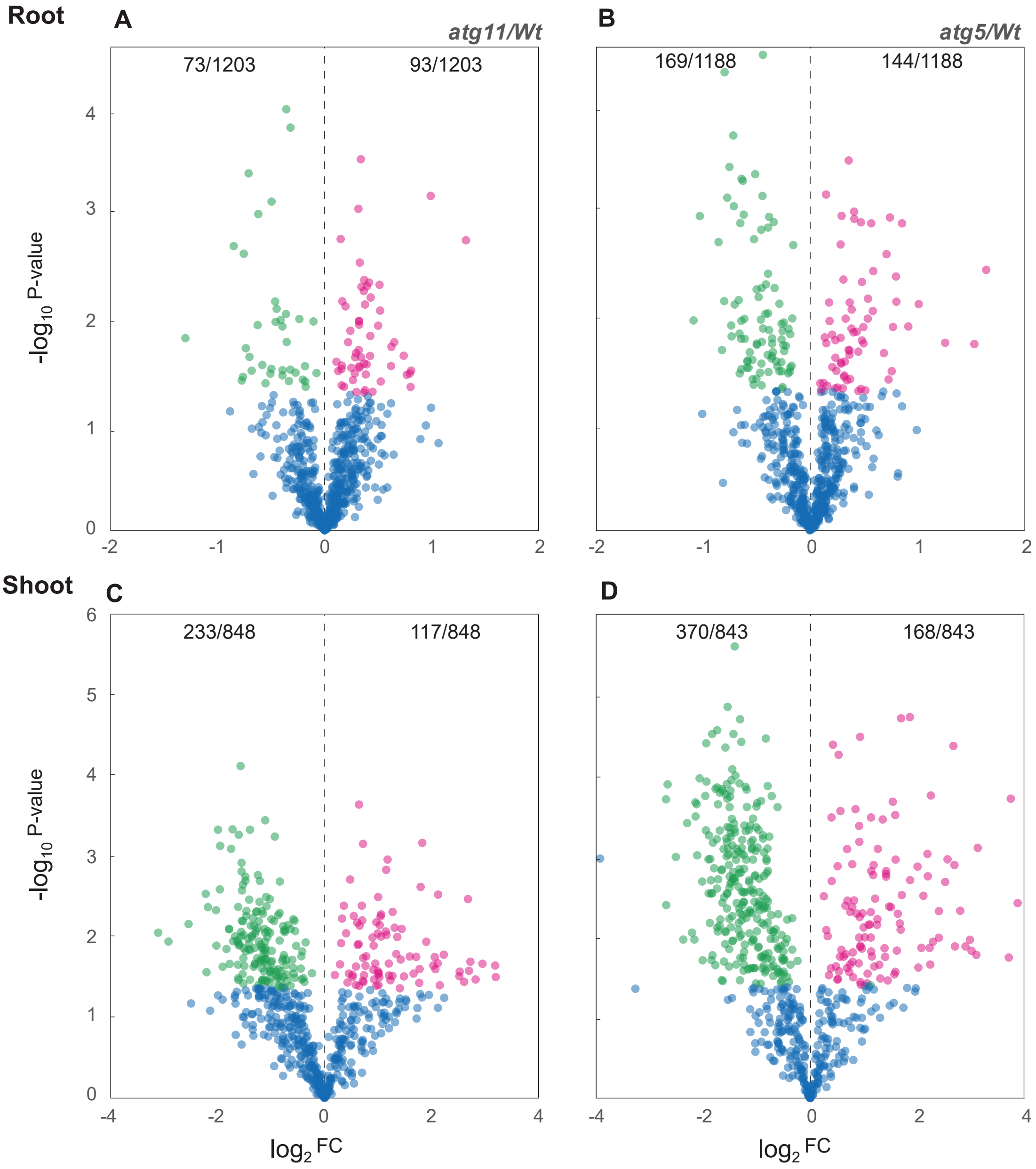

Supplemental Figure S4. Changes in protein abundance by label free quantification (LFQ) in roots and shoots of Arabidopsis autophagy mutants.
(Supports Figure 1.)
Volcano plots of log2 fold change (FC) in protein abundance values between mutants and wild type roots (**A,B**) and shoot (**C,D**). Proteins with significant changes in abundance (P-value<0.05, Student's T-test) were colored magenta (up) or green (down) for visualization. In *atg11*, 14% of root proteins show significant changes in abundance (8% up, 6 % down) (**A**) while 41% of shoot proteins show significant changes in abundance (14% up, 28% down) in comparison with wild type (**C**). In *atg5*, 26% of root proteins show significant changes in abundance (12% up, 14% down) (**B**) while 64% of shoot proteins show significant changes in abundance (20% up, 44% down) in comparison with wild type (**D**). The full list of all proteins identified and quantified is shown in **Data S1**.

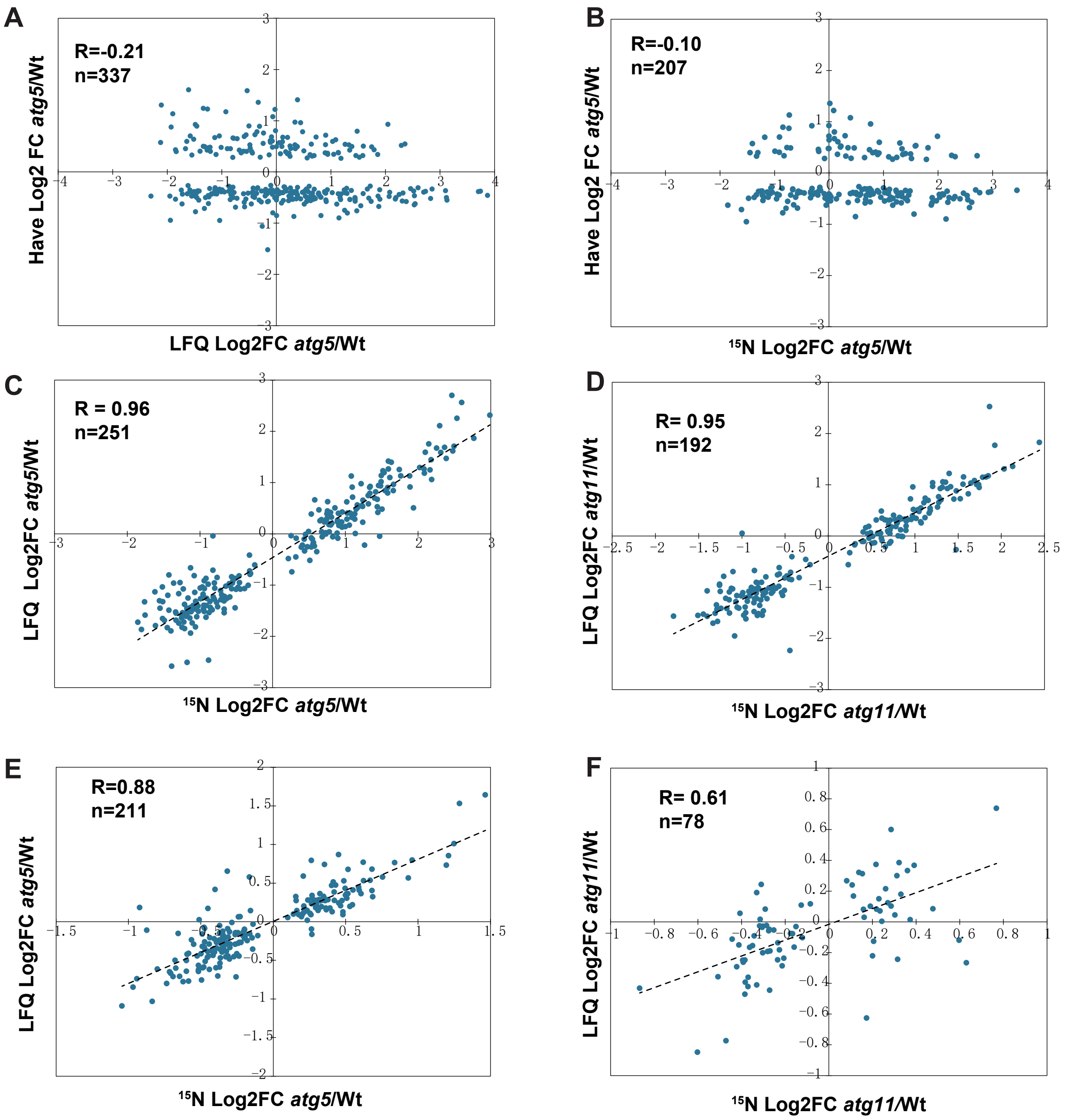

Supplemental Figure S5. Correlation analysis of protein abundance acquired from ^{15}N spike-in and label free quantification (LFQ) strategies.
(Supports Figure 1.)
Log transferred fold changes in protein abundance between *atg5* and wildtype Arabidopsis shoot from either label free quantification (LFQ) or ^{15}N spike-in methods were compared with the same Log transferred fold changes from Have et al., **New Phytologist**,2019 (ANOVA and Tukey tests, P<0.05 in Have study) (**A,B**). Log transferred fold changes in protein abundance in *atg5* and *atg11* mutant lines (Student's T test P<0.05 in ^{15}N spike-in) were compared between LFQ and ^{15}N spike-in methods in shoot (**C,D**) and root tissues (**E,F**). Correlation R values and the number of proteins for analysis were shown.

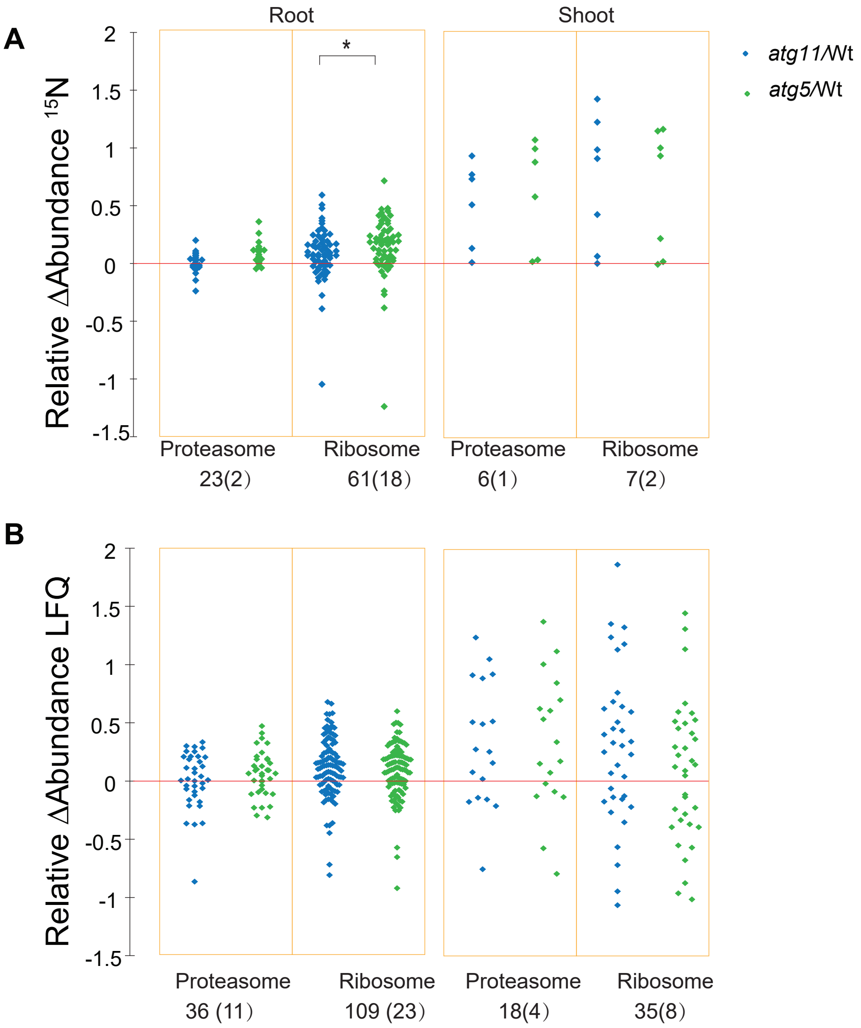

Supplemental Figure S6. Significant changes in abundance of ribosome and proteas-ome subunits inArabidopsis autophagy mutants.
(Supports Figure 1.)
Scattergrams of changes in protein abundance by ^{15}N spike-in (**A**) and LFQ (**B**)measure-ments in autophagy mutants in comparison to Wt are shown. 57-80% of ribosomal proteins and 55-78% of proteasome proteins showed a trend of abundance increase.Total number of quantified proteins in both mutant lines and the number with significant changes (Student's T-test, P<0.05) in at least one of the mutant lines were shown. Two-sample Kolmogor-ov-Smirno test was utilized to show differences in ribosome and proteasome relative Δabun-dance distributions in *atg11* and *atg5* compared with *Wt* (** P<0.01, *P<0.05).

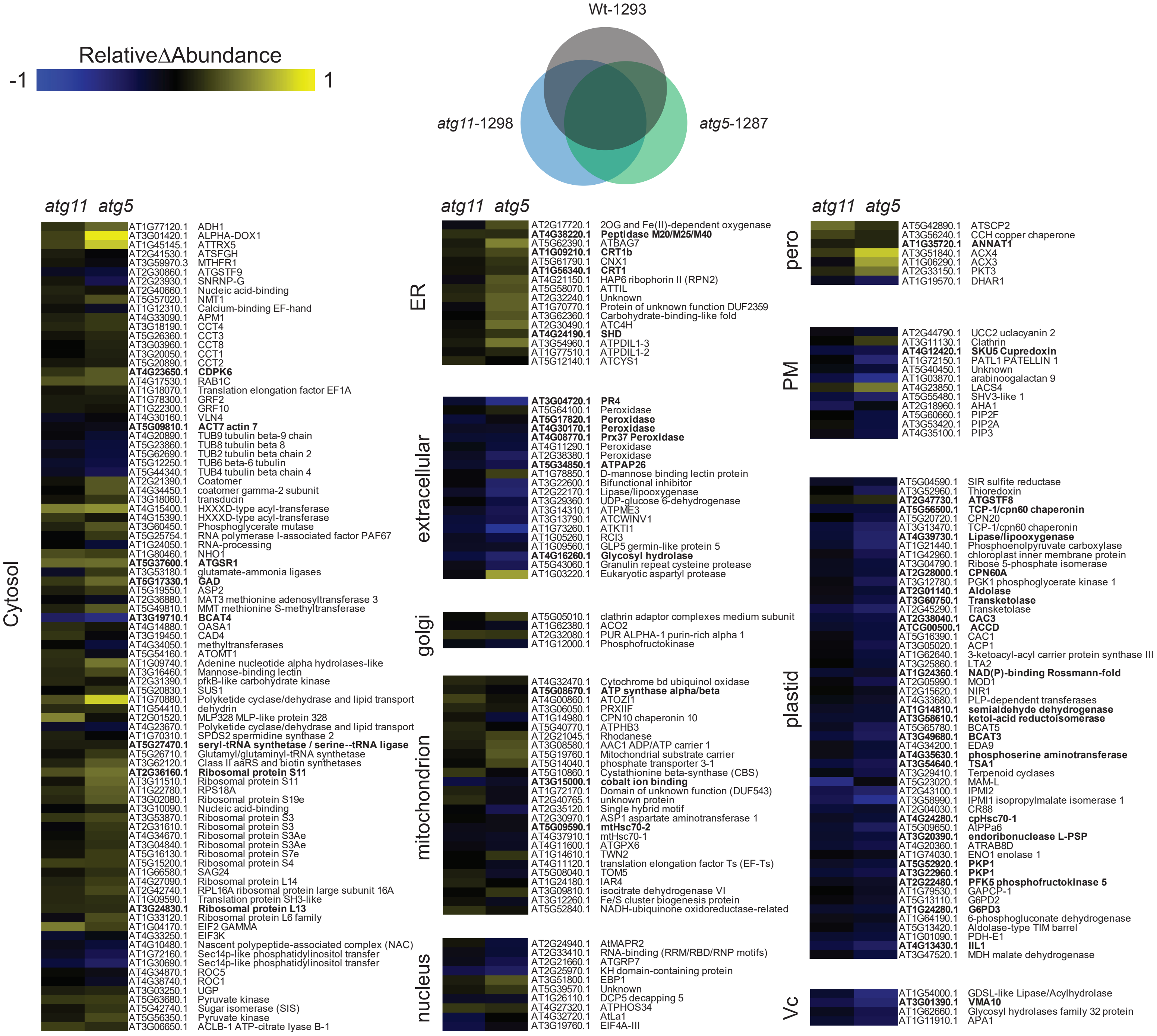

Supplemental Figure S7. Significant changes in relative Δabundance of 241 root proteins in Arabidopsis autophagy mutants.
(Supports Figure 1.)
Heatmaps are used to show the relative Δabundance of specific root proteins that are significantly different in abundance between an autophagy mutant and Wt. 44 proteins with statistically significant changes in abundance in both *atg5* and *atg11* are highlighted in bold font. Proteins are grouped into the subcellular locations in which they reside.

Supplemental Data. Li et al. (2022). Plant Cell.

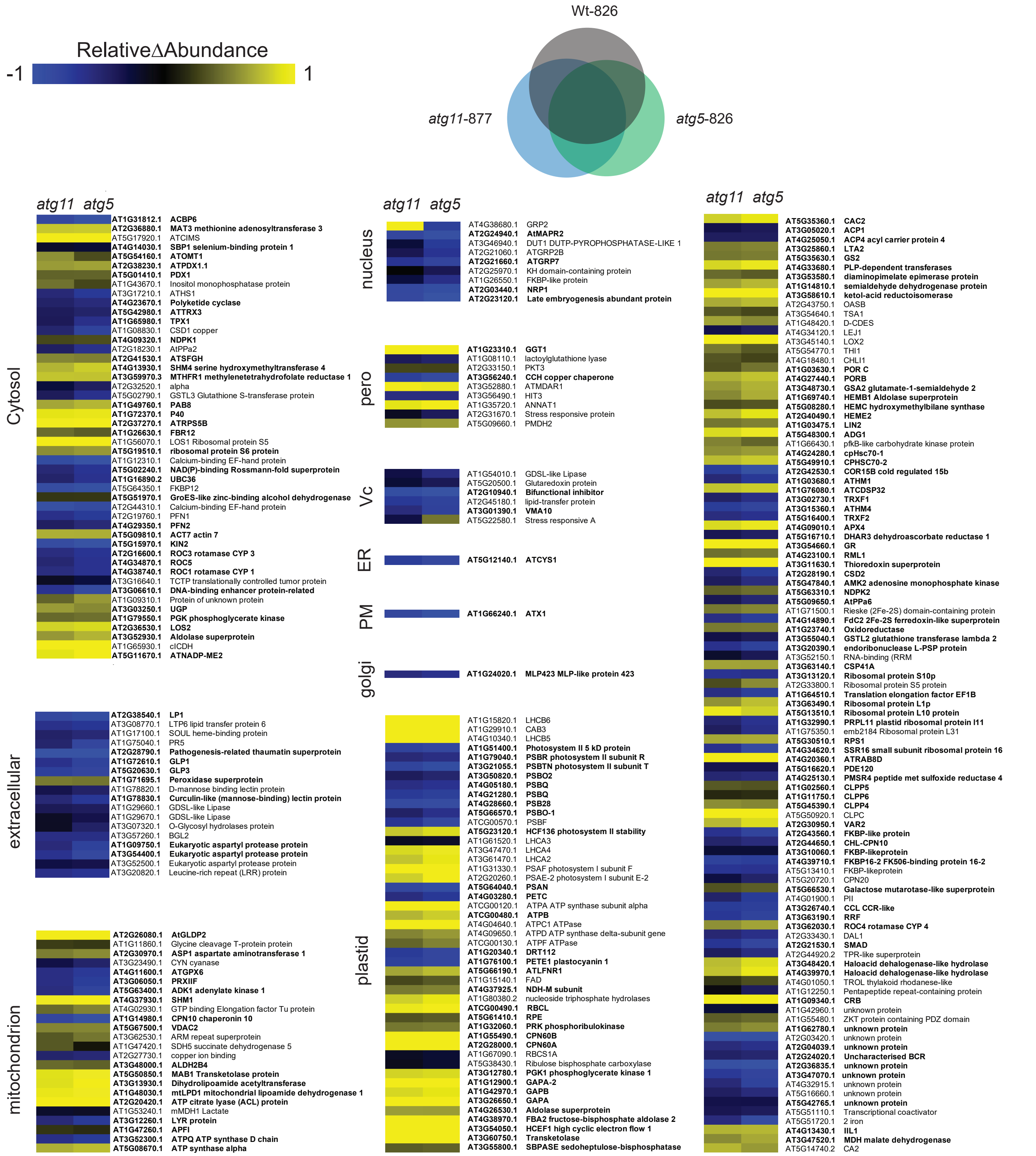

Supplemental Figure S8. Significant changes in relative Δabundance of 265 shoot proteins in Arabidopsis autophagy mutants.
(Supports Figure 1.)
Heatmaps are used to show the relative Δabundance of specific shoot proteins that are significantly different in abundance in autophagy mutants vs Wt. The 173 proteins with statistically significant changes in abundance in both *atg5* and *atg11* are highlighted in bold font. Proteins are grouped into the subcellular locations in which they reside.

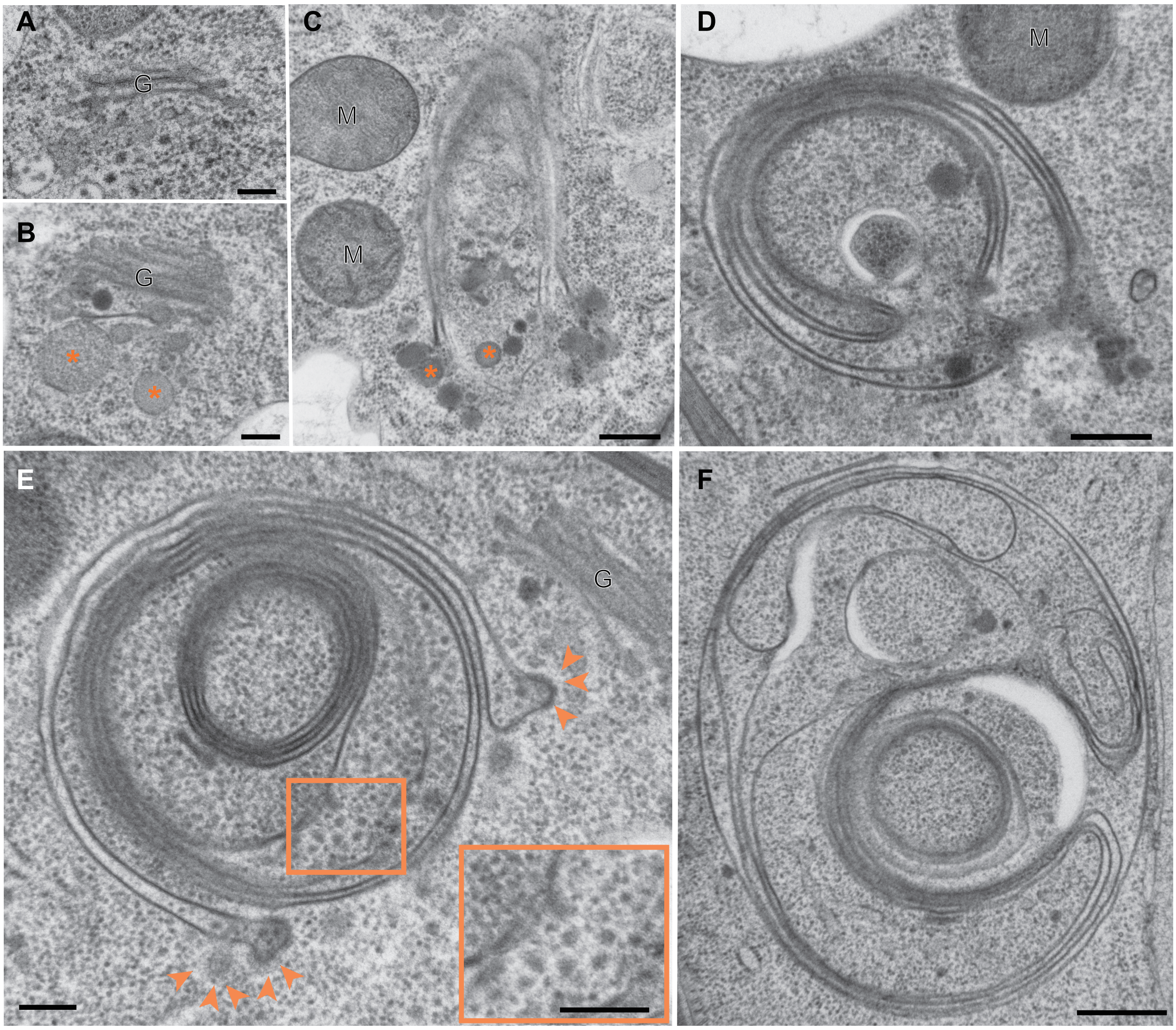

Supplemental Figure S9. Abnormal organelles observed by microscopy in *atg5* root cells. (Supports Figure 2.)

(**A**) Golgi stack in WT cells. (**B**) Golgi stack and associated TGN with dilated vesicle profiles (asterisks) in *atg5*. (**C**)-(**F**) Large membranous structures with concentric membranes in *atg5*. Some of these structures displayed budding profiles at their edges (asterisks in **C**). Budding sites with assembled coats (**E**, asterisks) are commonly seen on these structures. Enclosed by these membranes, there are electron dense aggregates 2-3 times larger than a cytosolic ribosome (**E** and inset is enlargement of the boxed region). G, Golgi stack; M, mitochondrion. Scale bars= 200 nm (**A**,**B**, **E**); 400 nm (**C**,**D**); 500 nm (**F**).

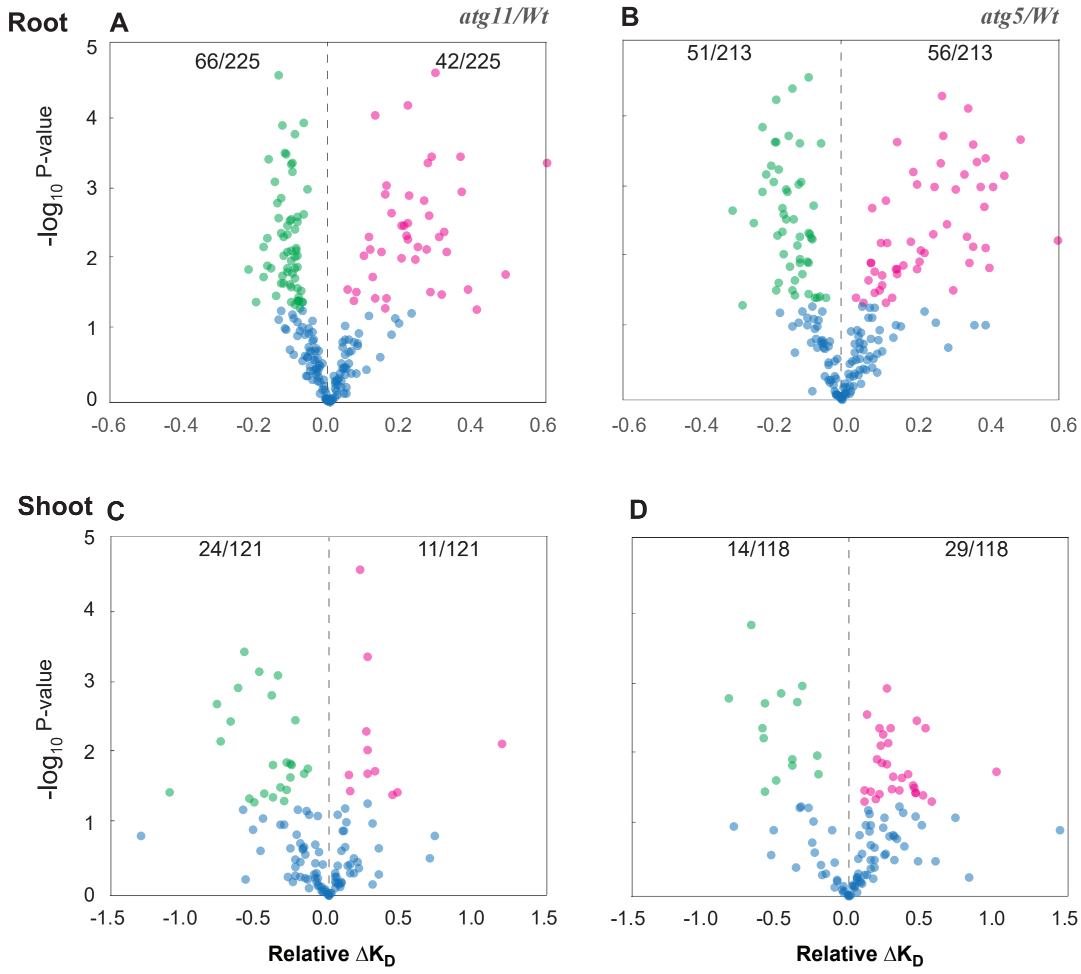

Supplemental Figure S10. Changes in protein degradation rate (K_D) in roots and shoots of Arabidopsis autophagy mutants compared to *Wt*.
(Supports Figure 3-5.)

Volcano plots **of** relative change in K_D values (ΔK_D) of each mutant compared to Wt for roots (**A,B**) and shoots (**C,D**) are shown. Proteins with significant changes (P-value<0.05, Student's T-test) were colored magnenta (increased) or green (decreased) for visualization. In *atg11*, 48% of root proteins show significant change in K_D (19% increased, 29% decreased) (**A**) while 29% of shoot proteins show significant changes (9% increased, 20% decreased) in comparison with wild type (**C**). In *atg5*, 46% of root proteins show significant changes in K_D (23% increased, 22% decreased) (**B**) while 37% of shoot proteins show significant changes (25% increased, 12% decreased) in comparison with wild type (**D**). The full list of all proteins is shown in **Data S3**.

Supplemental Data. Li et al. (2022). Plant Cell.

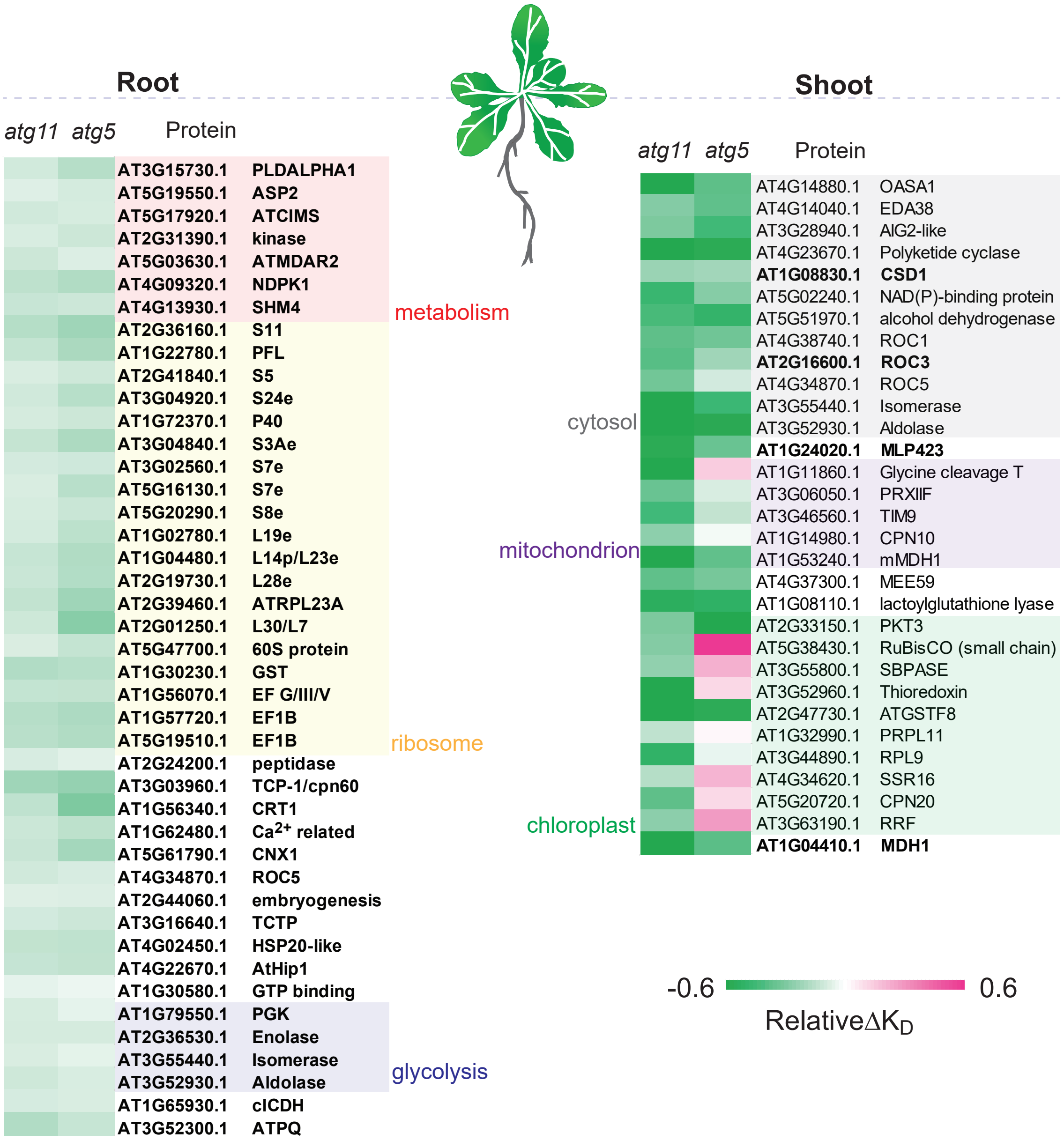

Supplemental Figure S11. Specific proteins that degrade more slowly in *atg5* and *atg11* roots and shoots compared with wild-type Arabidopsis.
(Supports Figure 3-5.)
(**A**) A heatmap of 43 root proteins with significantly slower degradation rate (relative ΔK_D) in both atg5 and atg11. (**B**) A heatmap of 31 shoot proteins with significantly slower ΔK_D in *atg5* or *atg11*. Proteins with significance differences in both *atg5* and *atg11* and shown in bold font. Proteins are grouped according to the top three functional categories in root and top 3 organelles in shoot tissues. Specific protein degradation rates in WT, *atg5* and *atg11* can be found in **Data S3**.

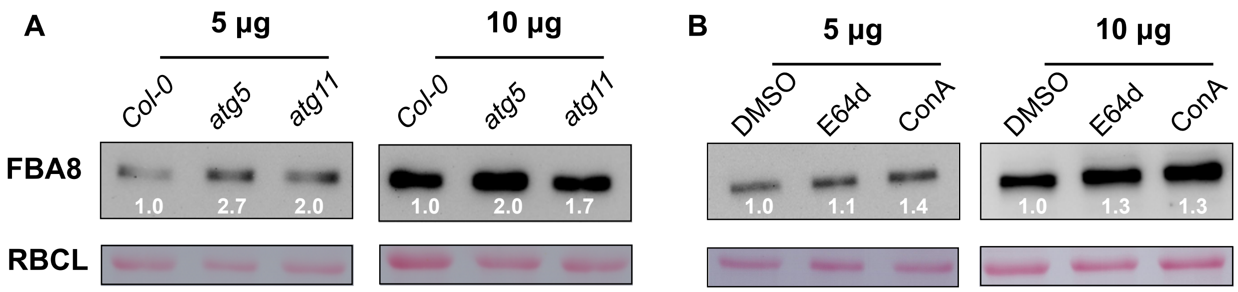

Supplemental Figure S12. Arabidopsis glycolytic FBA8 accumulated in *atg5* and *atg11* mutant lines and its degradation was inhibited by E64d and ConA treated wild type. (Supports Figure 6.)

FBA8 protein abundance was determined in Arabidopsis seedling Col-0 and mutant lines (**A**) with Col-0 treated with DMSO, 20 μM E64d and 1 μM ConA for 12 hours (**B**). Ponceau stained RuBisco large subunit (RBCL) was used for total protein equal loading control. Values of normalized FBA8 to Col-0 for mutant lines or treatment to DMSO control were shown.

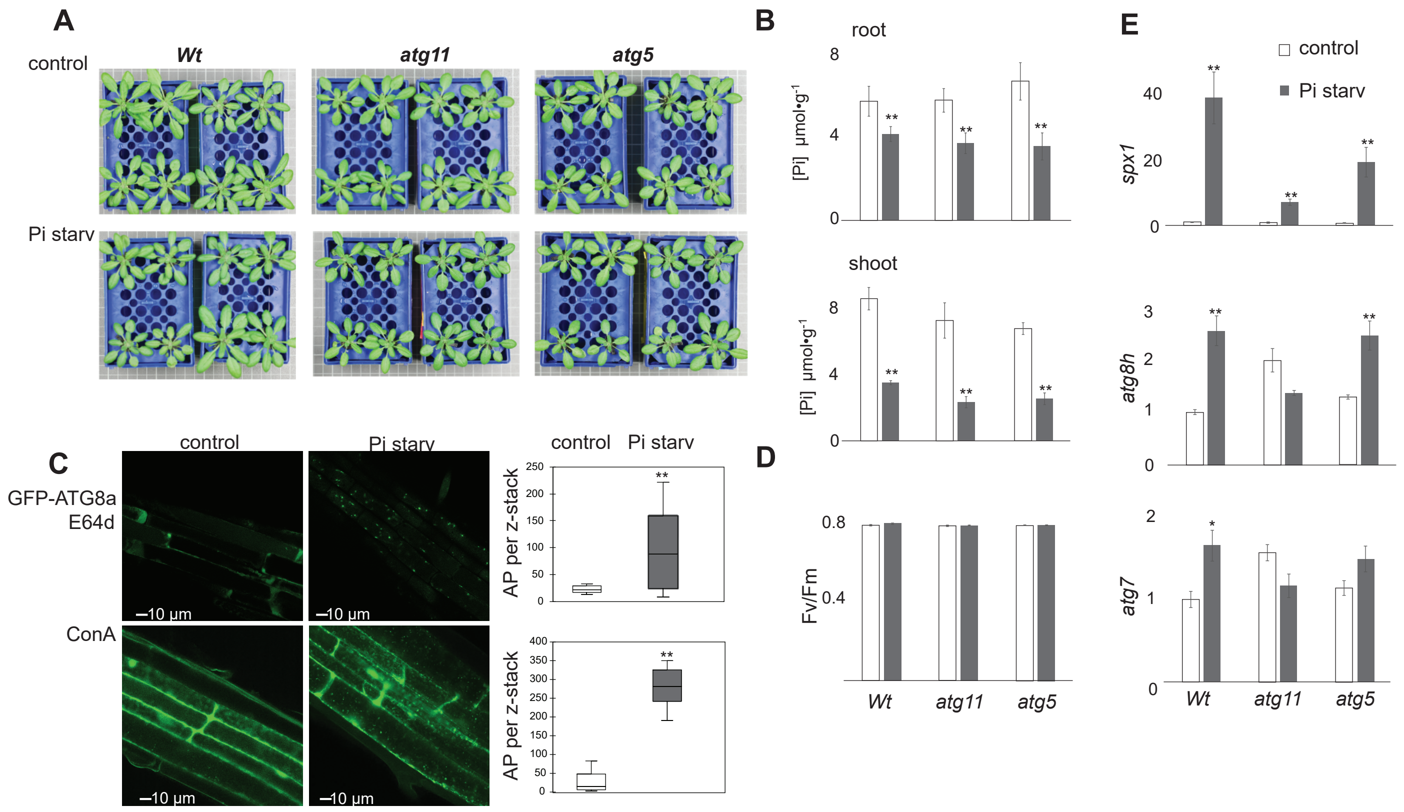

Supplemental Figure S13. Pi limitation induces changes in *atg5*, *agt11* and WT Arabidopsis plants. (Supports Figure 7-9.)

(**A**) Arabidopsis plants grown in hydroponics for 21 days were transferred to fresh growth media with/without Pi for three days. (**B**) Free inorganic Pi concentration per fresh weight of root and shoot tissues was measured by a colorimetric assay method. (**C**) Root tips of a *GFP-ATG8a* line under control and Pi starvation conditions were treated with E64d or ConA overnight before confocal imaging. Box plots indicate the median (center lines), interquartile range (borders of boxes) and minimum and maximum values (whiskers). Number of autophagic organelles (AO) per z-stack in roots grown under control or Pi limitation conditions (Kolmogorov-Smirnov two distribution test, **P<0.01). (**D**) Shoot tissue quantum efficiency of photosystem II (Fv/Fm) in *Wt*, *atg5* and *atg11* lines. (**E**) Transcript abundance of *SPX1*, *ATG8H* and *ATG7* in *Wt*, *atg5* and *atg11* under both control and Pi starvation conditions. Student's T-test, *P<0.05, **P<0.01. Error bars show standard deviations of four biological replicates.

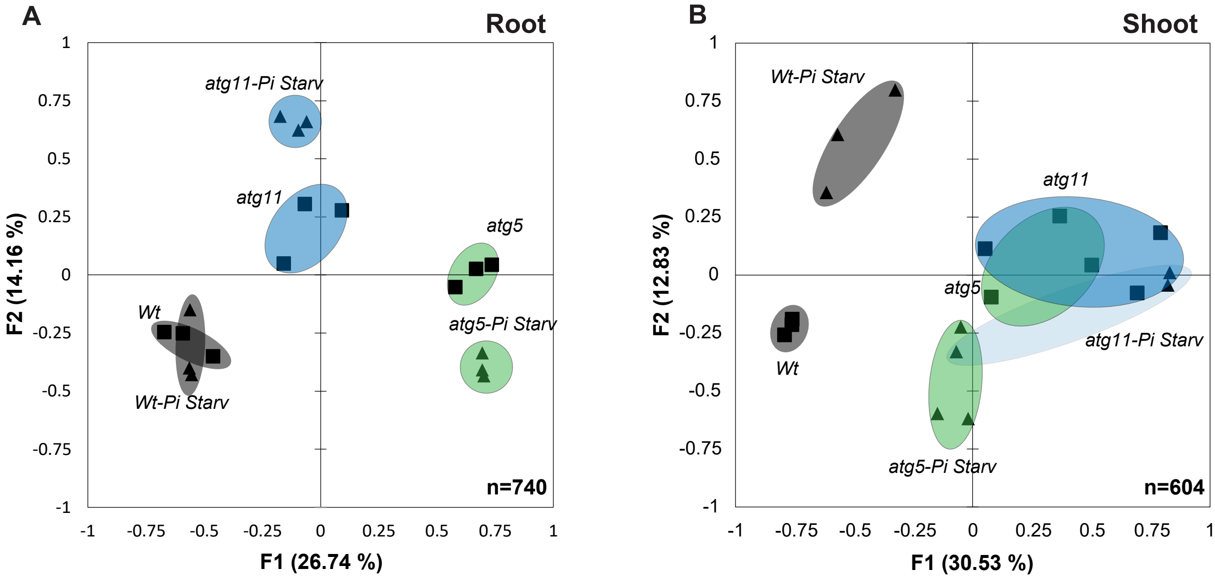

Supplemental Figure S14. A PCA to evaluate Pi limitation on label free quantification (LFQ) in *atg5*, *atg11* and *Wt*.
(Supports Figure 8-9.)
Protein abundance measurements using label free quantification (**A,B**) were first normalized and used for PCA. The number of proteins with measured abundance in all samples were shown in each graph. The full list of all proteins identified and quantified are shown in **Data S7**.

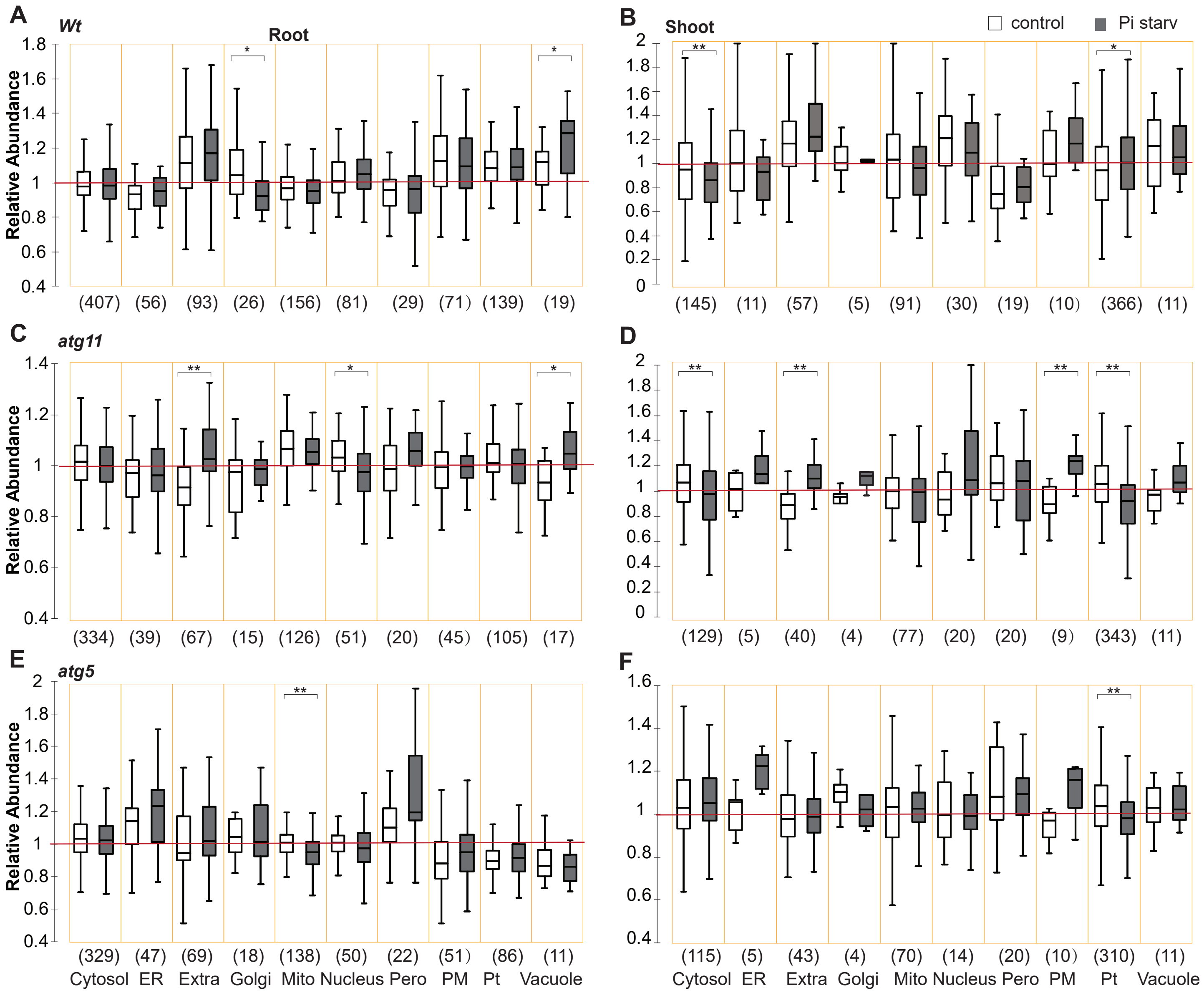

Supplemental Figure S15. Effects of Pi limitation on protein abundance in roots and shoots of Wt and autophagy mutants.
(Supports Figure 8-9.)

Box-plots are shown of the relative abundance of 1077 root proteins (**A**) and 745 shoot proteins (**B**) in wild type (Wt), 819 root proteins (**C**) and 658 shoot proteins (**D**) in *atg11*, and of 821 root proteins (**E**) and 602 shoot proteins (**F**) in *atg5* in response to Pi limitations. Box plots indicate the median (center lines), interquartile range (borders of boxes) and minimum and maximum values (whiskers). Protein groups are based on the subcellular location in which they reside. Box plots indicate the median (center lines), interquartile range (borders of boxes) and minimum and maximum values (whiskers). Two-sample Kolmogorov-Smirno test was utilized for comparison of control and Pi limitation distribution in each subcellular location to evaluate the effect of Pi limitation (** P<0.01, *P<0.05).

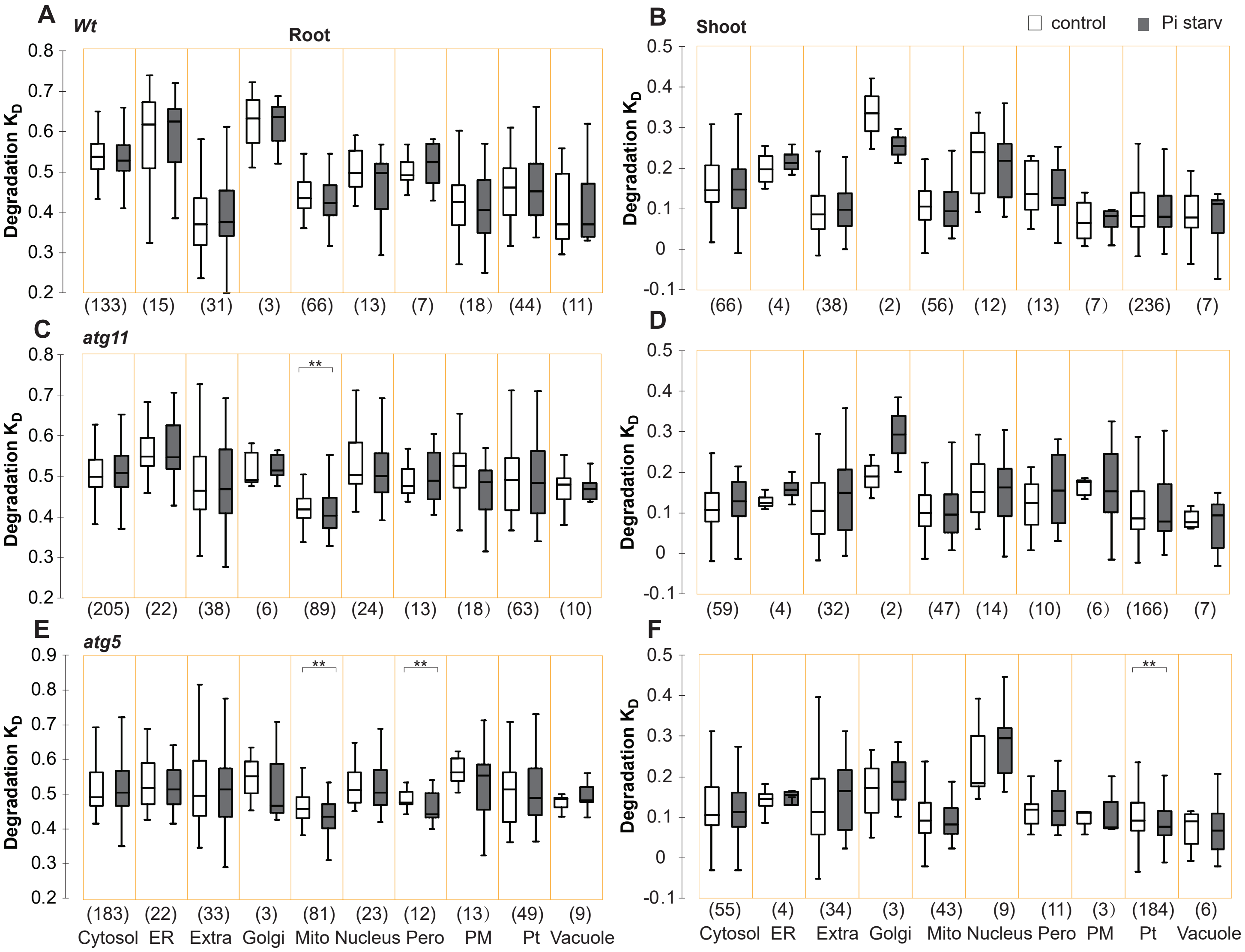

Supplemental Figure S16. Effects of Pi limitation on protein degradation rates in roots and shoots of *Wt* and auto-phagy mutants.
(Supports Figure 8-9.)

Box-plots are shown of protein degradation rates of 341 root proteins (**A**) and 348 shoot proteins (**B**) in wild type (Wt), 488 root proteins (**C**) and 347 shoot proteins (**D**) in *atg11*, and of 428 root proteins (**E**) and 352 shoot proteins (**F**) in *atg5* in response to Pi limitations. Box plots indicate the median (center lines), interquartile range (borders of boxes) and minimum and maximum values (whiskers). Protein groups are based on the subcellular location in which they reside. Box plots indicate the median (center lines), inter-quartile range (borders of boxes) and minimum and maximum values (whiskers). Two-sample Kolmogor-ov-Smirno test was utilized for comparison of control and Pi limitation distribution in subcellular location to evaluate the effect of Pi limitation (** P<0.01, *P<0.05).

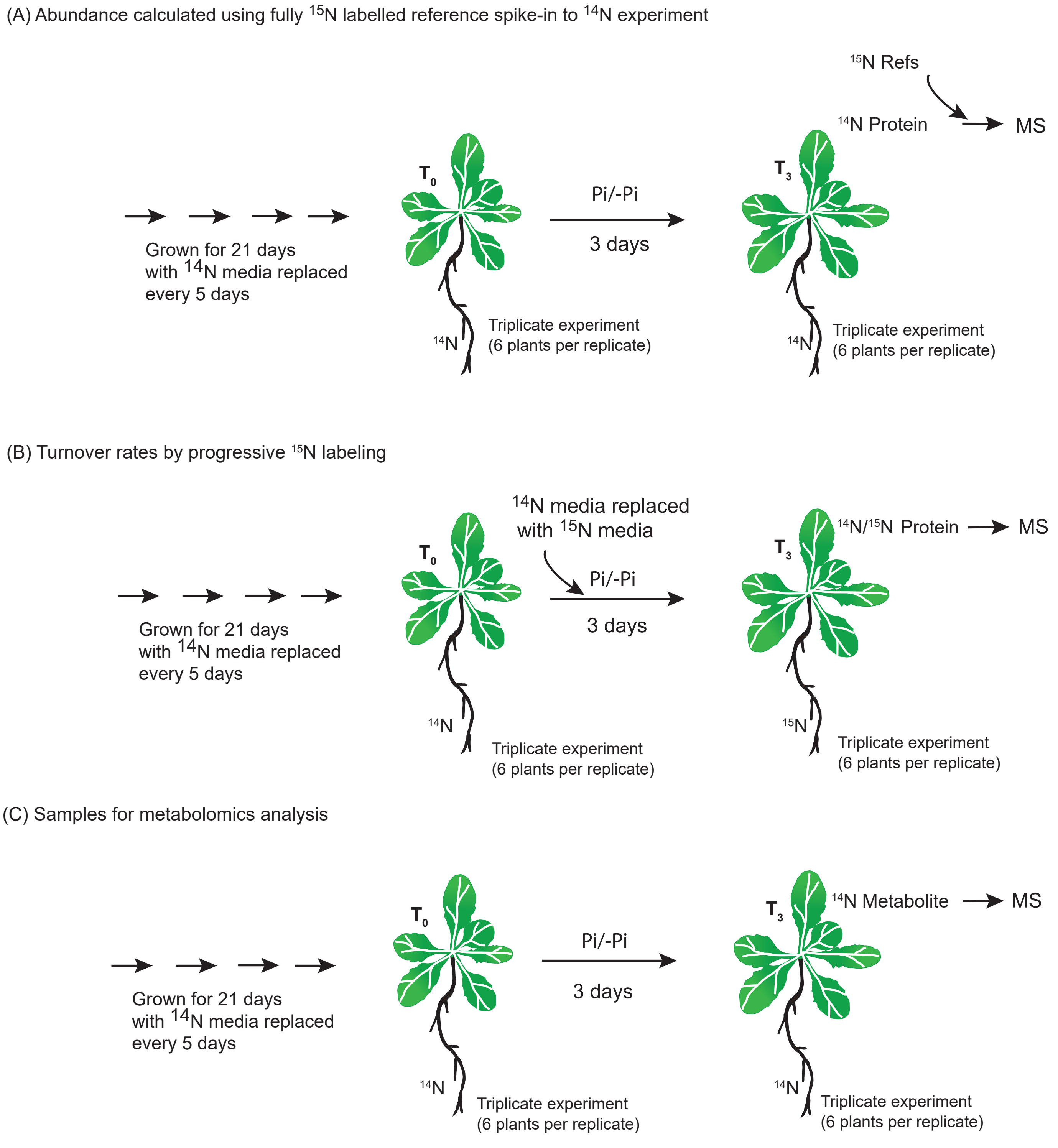

Supplemental Figure S17. Workflow of analysis to determine protein abundance, protein degradation rates and metabolite abundances in samples from Arabidopsis plant tissues. (Supports Figure 1, Figure 3-5, and Figure 7-9.)

(**A**) Relative protein abundance of specific proteins was determined by adding a spike-in ^{15}N labelled reference sample to each unlabelled experimental sample before preparation of peptides for MS. (**B**) Protein degradation rate of specific proteins was determined by progressive ^{15}N labelling *in vivo* before preparation of peptides for MS. (**C**) Metabolite profiles were determined by extracting from samples collected from plants grown in unlabeled growth media before preparation of metabolites for MS.
