# Supplementary material for: Defects in autophagy lead to selective in vivo changes in turnover of cytosolic and organelle proteins in Arabidopsis
Source: Plant Cell. 2022 Jun 29;34(10):3936–60. doi: 10.1093/plcell/koac185 (PMC9516138; doi:10.1093/plcell/koac185)
Supplement: koac185_Supplementary_Data [file tpc21.00373.PeerReview.pdf › koac185_Supplementary_Data/tpc21.00373.PeerReview.pdf]

Defects in autophagy lead to selective *in vivo* changes in turnover of cytosolic and organelle proteins in Arabidopsis

Lei Li, Chun Pong Lee, Xinxin Ding, Yu Qin, Akila Wijerathna-Yapa, Martyna Broda, Marisa S. Otegui, A. Harvey Millar

Corresponding author: Lei Li (lei.li@nankai.edu.cn) and A. Harvey Millar (Harvey.millar@uwa.edu.au)

Review timeline:

**TPC2021-RA-00373**	Submission received:	April 28, 2021
	1st Decision:	June 16, 2021 *revision requested*
**TPC2021-RA-00373R1**	1st Revision received:	January 1, 2022
	2nd Decision:	February 10, 2022 *revision requested*
**TPC2021-RA-00373R2**	2nd Revision received:	May 9, 2022
	3rd Decision:	May 30, 2022 *acceptance pending, sent to science editor*
	Final acceptance:	June 21, 2022

REPORT: (The report shows the major requests for revision and author responses. Minor comments for revision and miscellaneous correspondence are not included. The original format may not be reflected in this compilation, but the reviewer comments and author responses are not edited, except to correct minor typographical or spelling errors that could be a source of ambiguity.)

TPC2021-RA-00373 1st Editorial decision – *revision requested* June 16, 2021

We have received reviews of your manuscript entitled "Defects in autophagy lead to selective in vivo changes in turnover of cytosolic and organelle proteins in Arabidopsis." Thank you for submitting your best work to The Plant Cell. The editorial board agrees that the work you describe is substantive, falls within the scope of the journal, and may become acceptable for publication, pending revision and potential re-review.
As you will see from the reviewer's comments below, there is enthusiasm for your study, but also concerns that would need to be addressed prior to publication. These include concerns over whether the method used can reveal turnover rates, but also relate to the technical and biological validation of the data presented. These concerns seem pertinent, and we would expect you to address these in your revision. If there are suggestions that you consider unfeasible, please detail this in your response to reviewers. A note from the Editor-in-Chief: We are trying to make a concerted effort to change green/red comparisons to green/magenta to make our figures understandable to those with color vision deficiencies. We noticed that a small number of your figures utilize red/green contrasts, so as you prepare the final version of the figures, please check the figures for red/green color use. Magenta is a good substitute for red, even if the data are labeled as "RFP" or "mCherry" - readers will understand that colors can be changed, and indeed, it can be adjusted relatively quickly by using the "hue" setting in Photoshop or similar software. For example, the following figures may need to be corrected: Figure 2D to 2H - the bars in red and green shading may be hard to discern, Figure 4 - the heat map, Figure 5 - all panels, maybe Figure 8G (I don't see much green), Figure 9G; you might want to check over the Supplemental Figures for similar comparisons. Please note that color changes do not need to be highlighted or tracked in the revised manuscript, but could be noted in the cover letter or response document. -- Reviewer comments:

TPC2021-RA-00373R1 1st Revision received January 1, 2022

Reviewer comments and **author responses**:

Reviewer #1:

Autophagy is a major degradation pathway that is crucial for a plethora of stress responses. In this manuscript, the authors performed heavy isotope labeling proteomics to determine the degradation rates of proteins in two different

autophagy mutant backgrounds in two different organs (root/shoot) and under phosphate starvation. Overall, the manuscript is nicely written and the data are well presented. However, I am not convinced that the authors are doing justice to their datasets by not performing any confirmatory experiments for their exciting leads. As the authors also mention, similar omics experiments have been performed in autophagy mutants before. They propose that their approach to measuring degradation rates is more accurate, but as readers, we can't judge this, since the only measures we are seeing are a list of proteins and plots. I think the confirmatory experiments that I am asking are different than asking people to validate RNA-seq results with qPCR. For me, the main issue is the lack of biology, not the technical validation. Please see below a list of detailed experiments that I think could improve the manuscript.

Point 1. One of the major issues of using atg mutant is the pleiotropic phenotypes that arise due to the lack of a major homeostatic pathway. As atg mutants keep accumulating garbage, this leads to increased cellular stress levels and induces a variety of stress responses, including the salicylic acid pathway (see papers from Masclaux-Daubresse lab). Even though the authors have checked that the age they use (21-day old plants) do not show senescence, they can't be sure that they are observing cellular stress. For example, the chaperone complex they are identifying could have higher levels in the atg mutant to help to fold the aggregated proteins that accumulate in the atg mutants. If they have evidence showing this is not the case, they should make it clear to the readers.

RESPONSE: We agree with the reviewer that autophagy defects leads to protein accumulation in specific organelles and functional groups. This was evident from our data shown in Fig 1, but a large proportion of these proteins that accumulation are not due to direct autophagy degradation (see new Fig 3). Chaperone accumulation as noted, could be a consequence of a cellular stress stimulated rescue response involving enhanced transcription and follow-up protein synthesis as the reviewer proposed. If this hypothesis was correct, we would expect these chaperones (CCTs) to have a fast turnover rate due to rapid synthesis. However, our turnover analysis shows quite the opposite. CCTs overall have a slower turnover rate and accumulate in autophagy mutants (new Fig 5, DataS6). This supports CCTs being potential autophagic cargo. We presented protein turnover rates compared to protein abundance to directly address the reviewer's concerns.

Point 2. I would have expected at least a list of "usual suspects" such as ATG8 isoforms or NBR1 to over accumulate in the atg mutant. If this is the case, it would be nice to have a supplemental table for known autophagy cargoes.

RESPONSE: We don't observe ATG8s or NBR1 in our proteome, probably due to their relative low abundance. We do appreciate the reviewer's suggestions to show more clearly how our data relates to known autophagy cargos. We have thus made a new Fig 4-'known autophagy targets' and new Fig 5-'putative autophagy targets' from our data (originally Table S1 and S2) to show protein abundance and turnover rate patterns from our data alongside five reference studies of ATG8 interactors and/or proteomics in autophagy mutants. Known autophagy targets include subunits of the ribosome, proteasome, ER, mitochondrion, chloroplast and peroxisome. Putative autophagy targets include components of glycolysis, T-complex, metabolism, cytosol and others.

Point 3. As I mentioned above, for one substrate-such as the chaperonin complex, they should show, a) it is recruited to the autophagosomes- colocalization/coIP, b) it undergoes autophagic degradation-using flux assays.

RESPONSE: We appreciate this request. To satisfy it, we have chosen to use the glycolytic enzyme Fructose Bisphosphate Aldolase (FBA8) as a case study to validate our claim. Firstly, we constructed a FBA8-DsRed chimeric and transform it into protoplasts prepared from a stable Arabidopsis line expression ATG8a-GFP. FBA8-DsRed was observed to be internalized into autophagosomes upon application of the TOR kinase inhibitor AZD8055 to activate autophagic flux. Secondly, using FBA8 antibodies, we show it accumulated after treatment of wild type plants with the autophagy degradation inhibitors E64d and concanamycin. These two independent experiments support our combined protein abundance and turnover rate analysis. We have made a new Fig 6 and Fig S9 to show these results.

Point 4. Similarly, they need to validate at least one of the atg5 and atg11 specific cargoes using the routine autophagy assays that are well-established in the PIs labs.

RESPONSE: We chose the glycolytic enzyme FBA8 as a case study to validate our claim as mentioned above. To verify specific roles of ATG11 and ATG5 in specific cargo is a large comprehensive endeavor beyond the aim of this manuscript, but possible in future studies.

Point 5. They are detecting only a subset of organelle proteins in their cargo sets. Does this mean, piecemeal autophagy? or there is a technical issue of not having enough depth in the proteome? In either case, this needs to be discussed clearly.

RESPONSE: While we observe similar abundance and degradation changes for a range of ribosomal and proteasomal subunits in mutants that support them to be autophagy targets as a whole, organelles such as mitochondria tended to show putative autophagy cargos in only the electron transport chain or TCA cycle proteins (New Fig 4). However, we hesitate to make comment on piecemeal autophagy due to the nature of this study using total cellular proteomes and the heterogeneity of organelles. An in-depth investigation of all proteins in isolated organelles with a higher level coverage would be needed to help determine if piecemeal or engulfment of whole organelles is likely to be occurring. We now raise this issue in the discussion noting these limitations in the current study.

Point 6. In Fig6- the microscopy needs to be presented with and without E64D. It is very challenging to quantify autophagic bodies in the vacuole and they should see an increase in the number of autophagosomes in the cytosol.

RESPONSE: No autophagic bodies in vacuole could be detected using DMSO as a control (New Fig 6). But we do understand that E64D leads to accumulation of ATG8 associated autophagic bodies and also that ATG8 independent vesicles have been reported (Goto-Yamada et al., FIPS, 2019). So we added a concanamycin treatment in the figure which gave a similar result to the Pi starvation activated autophagic bodies accumulation observed in the vacuole (New Fig S10). The fact that Pi starvation can activate autophagy has also reported in both yeast and plants (Tasaki et al., 2014; Yokota et al., 2017; Naumann et al., 2019).

Reviewer #2:

This manuscript by Li et al. entitled "Defects in autophagy lead to selective in vivo changes in turnover of cytosolic and organelle proteins in Arabidopsis" attempted the laudable task of carefully defining how autophagy impacts the turnover rates of proteins using Arabidopsis as a model. Through comparisons of 15N- and 14N-labeled proteins from WT and two atg mutants (atg5 and atg11), they hoped to identify proteins that were more abundant in the atg mutants versus WT under both non-stressed and phosphate-deficient conditions, which might represent autophagy targets. We completely concur that measurement of protein turnover for individual proteins are needed to fully understand the impact of autophagy on plant proteomes and that trying to determine these rates en masse would be the best strategy. Unfortunately, we do not believe that the authors have provided convincing evidence that their approach was successful, nor present their story in an approachable and rigorous way to warrant publication at this time.

Point 1. 1) The use of 15N labelling is an excellent approach, but the measurements made were really not a determination of rate but really a one-point assay. From what we understand, the authors actually compared the 14N/15N ratio for individual peptides when they mixed unlabeled atg mutants containing 14N or pulsed for 3 days with 15N (progressive labeling) with WT plant grown continuously with 15N. Can an accurate rate be measured from such single timepoint of 3 days. Estimates of global protein turnover rates in plants are around 2-3 days, so with a labeling that proceeded for only 3 days, the authors might have actually labeled only the faster synthesized pool. It seems that authors really needed to do the pulse labeling over many time points. While we realize that approach adds substantial duty time to the MS, it is really important.

RESPONSE: For clarification, we did not mix ^{15}N progressive labelling samples with any 15N fully labelled samples. We only spiked in ^{15}N fully labeled WT as a reference to unlabeled WT, atg5 and atg11 mutants for protein abundance measurements. We measured protein abundance and protein turnover separately as illustrated (New Fig S13). To address the validity of single time point measurement of protein turnover rates we would like to outline the following. A metabolic labelling coupled with the mass spectrometry strategy can determine protein turnover rate with a single time point of sampling and measurement. Such a single time point labelling experiment measures the average degradation rate value between that time point and the beginning of labelling. This was reported in mammals (Schwanhausser et al., Nature, 2011). In the past decade, we have developed a time point protein turnover analysis strategy in plants (Li et al., MCP, 2012, Nelson et al., Plant Physiology, 2014 Li et al., Plant Journal, 2017, Li et al., Plant Cell, 2017). All these studies support that single time points turnover over measurements produce the same rate as protein turnover rates measurements over a time course under steady state conditions. We thus designed our turnover analysis with special care to use exactly the same steady condition we had used previously

(Li et al., Plant Cell, 2017). We have now clarified this in the methods section. The 3 days of labelling was chosen because it can measure both relatively fast and slow protein turnover rates. Classic rapid turnover proteins like GRP7 (~0.6 d^{-1}) and Nascent polypeptide-associated complex (NAC) alpha subunit (~0.9 d^{-1}) are quantified in shoot and root tissues (Data S3). Moreover, the rates of slow turnover proteins in shoots (~0.0 d^{-1}) and roots (~0.2 d^{-1}) were also determined (Data S3). Our protein turnover measurements in this study and previous work (Li et al., PC, 2017) shows Arabidopsis leaf tissues have a median protein turnover rate of ~0.1 d^{-1}; equivalent to half-lives of 6~7 days. Root tissues have a faster protein turnover rate of ~0.5 d^{-1}; equivalent to 1~2 days. So the 3 days labelling is the best compromise to measure protein turnover rates for a major set of proteins in both shoot and root.

Point 2. At the very least, the authors could have shown that some of the proteins that they predicted are autophagy targets were actually degraded slower in the atg5 and atg11 backgrounds through detailed kinetic studies by MS. Moreover, it would have been nice to test turnover rates using another method besides MS, such as pulse labelling with 3H or 14C amino acids with and without cycloheximide to validate their approach.

RESPONSE: There is no available method to target just a few selected proteins for this type of shot-gun proteomics strategy of turnover rate. The current measurement of protein degradation rates provide an average rate between 21 and 24 days in wild type and atg5 and atg11 backgrounds. These average rates provides the information needed for the main claims in the manuscript. To measure protein degradation rate over multiple time points would only help if the purpose was to investigate protein degradation changes over different developmental stages. Pulse labelling with exogenous radioactive amino acids and cycloheximide treatment requires there to be no difference in rates of endogenous amino acid synthesis for the results to be interpreted, and evidence suggests autophagy itself alters amino acid pool sizes, so this method would not be a validation but an entirely different study needing new controls and assessments to be made. Further, 3H or 14C labelling can only tell whether a certain 'band' on a gel shows obvious changes, it can't be coupled to MS analysis directly. A band can contain multiple proteins that are hard to differentiate. We think that comparing our data in more detail to independent reports of roles in autophagy (New Figs 4 and 5 and Supplemental Data 6) and confirmatory protoplast transformation for co-localisation (new Fig 6) and the degradation assay (new Fig S9) for FBA8 serve as better means to support our findings from protein abundance and degradation measurements.

Point 3. It was unclear whether MS approach actually provided statistically meaningful results. No where in the paper did the authors show how their MS data compared among the biological replicates or indicated that technical replicates were performed in which the samples were analyzed by MS more than once. When we look at the volcano plots in Figure S2, it unclear whether the skew on the data points toward WT or the atg mutants are actually real changes or simply reflect the noise inherent in MS data. It was also unclear if the authors normalized their MS data among the samples (for example, atg5 versus atg11). We can also image that the separation of each sample into 96 fractions by HPLC and then combined into 12 pools could potentially contribute to noise. One way to test this notion is to compare 14N WT with 15N WT and see if similar skews are evident. Also compare the data from one biological replicate to the others. The seemly random collection of proteins identified by MS in the various samples (atg versus WT phosphate-rich versus starvation), and mentioned throughout the paper, are a potential result of this noise.

RESPONSE: We have performed and presented PCA analysis for the three biological samples of each genotype in Fig 8 and Fig 9. The results showed the biological replicates clustered separately for Wt, atg5 and atg11 in both root and shoot tissues with/without Pi starvation treatment (Fig8 and 9). So genotype based differences between the samples are clear. The protein abundance values were acquired from ratios compared with the same fully 15N labelled reference, they are not subject to the same noise attributed to other label free strategies. Protein turnover rates were acquired from ratios between 15N labelled and 14N non-labelled in the same sample. Compared with a typical label free mass spectrometry strategy, these values are not subject to the same variation in sample preparation such as HPLC separation and mass spectrometry status. Due to the quantification method used, we cannot directly compare 14N WT and 15N WT abundance; we measure a ratio. However, to ease the concerns raised, we now present a correlation matrix between biological replicates in Wt, atg5 and atg11 for protein abundance and turnover rates in root/shoot tissues (now supplied in Data S1 and Data S3). Fig S2 values are from Data S1 and Data S3. It is apparent that biological replicates from the same genotype show the highest correlation values. So we contend we can compare average values between atg5, atg11 and Wt. To further clarify this, we have made the new Fig 4 and Fig 5 to show the normalized protein abundance and degradation rates in every single biological replicate in Wt, atg5 and atg11 lines. We think this will help the reviewer and readers to judge the quality of the MS data and the biological claims made. In this study, we focus on the autophagy defects on protein

abundance and turnover changes rather than the techniques we already described in detail in previous publications. For protein abundance measurements we compare peptides to the same 15N spike-in reference and compared the normalized values across all the samples. For protein turnover measurements, we used a median-polish strategy to reduce random variation between biological samples. Both these strategies have been discussed in detail in a previous publication (Li et al., Plant Cell, 2017).

Point 4. As for analysis of the data, we are concerned that the small number of proteins identified among the MS runs limit the formation of statistically meaningful results when the data were parsed into various protein compartments. For example in Figure 1, can the authors really say anything about the turnover of Golgi proteins when only 4 and 1 in roots and shoots, respectively, showed significant results among the hundreds that exist in this compartment? See the PM and ER proteins in Figure 1 for other examples. Or ribosomes where on 2 of out 7 detected showed a change or 2 out of 23 proteasome subunits showed a change. This problem is then compounded by comparing the atg5 and atg11 backgrounds were the responses for some proteins and compartments differed among the two mutants. See the bar graphs in Figure 5C and D for examples. Maybe some of these differences reflect the phenotypic impact of the mutants or are just another example of noise. There is just not enough data to make strong conclusions about how an entire compartment or protein complex is responding. Lines 279 to 291 illustrate our point. In roots only one mitochondrial protein, 2 ER proteins and 12 cytosolic proteins were found to stabilized in the atg backgrounds. Could this muted response simply reflect noise in their analyses?

RESPONSE: Figure 1 shows proteins that differ in abundance comparisons between mutants and wild type. We agree with the reviewer that the small number of proteins with quantified abundance differences cannot serve to show specific patterns of change within a subcellular compartment. Distribution of proteins that significantly change in abundance into cellular compartments is aimed to help readers consider the spatial arrangement of the major protein abundance changes measured. To answer the further question of whether a specific cellular compartment or organelles showed general changes in abundance or selective losses requires a more in-depth investigation of protein abundance and protein turnover rates in purified organelles or enriched cellular compartments that cover most proteins in that location. That is beyond the aim of this study.

Point 5. When we look at the scatter plots in Figure 5A-D that correlated the relative abundance of the proteins in atg versus WT and the relative degradation rate, it was then unclear how much of this is caused by noise. It would have been very helpful to confirm whether some of the outliers really had different degradation rates or differences in transcription.

RESPONSE: We performed these scatter plots to show the overall correlation between degradation rate and abundance changes as a whole and in selected cellular compartments. We agree with the reviewer that showing protein degradation rates for specific proteins is helpful. We have now made the new Fig 4 and Fig 5 (all data shown in Data S6) to show normalized protein abundance and degradation rates of specific proteins in every single biological replicate in Wt, atg5 and atg11 lines. Readers can also find the raw data used to generate these numbers in Data S5.

Point 6. It is clear that the authors need to validate their results by other methods such as using Western blots to test various proteins in each of the compartments in the atg versus WT seedlings. This lack of depth also made the paper hard to comprehend as the authors tended to focus on the few proteins they identified as representative of an entire compartment.

RESPONSE: To partially address this issue, we used TEM counting of organelle numbers which tells us a tendency but not significant changes in amount. We don't think that western blotting with selected markers is proficient enough to test each of the compartments. To answer whether a specific cellular compartment or organelles showed general or selected changes really needs a more in-depth investigation of protein abundance and turnover of purified organelles or enriched cellular compartments that cover most proteins in that location, which is beyond the capabilities of this study. Instead, we provided new protoplast transformation (new Fig 6) and degradation assay (new Fig S9) using Western blotting for the glycolytic enzyme FBA8 to show the utility of our study to find putative autophagy targets that can then be independently confirmed. These new types of experimental evidence serve as better means to support our findings from protein abundance and degradation measurements.

Point 7. While the authors are correct that transcription based on mRNA abundances do not tell the complete story of protein abundances, they can help paint a more coherent picture. Toward this end, it would have been helpful to have included RNA-seq or qRT-PCR data of key examples.

RESPONSE: The limited correlation between transcription and protein abundance is widely accepted, so transcriptional studies would only partially aid the aims proposed. Moreover, such studies of protein abundance and transcription have already been performed. So rather than perform more, we now compare our data with sets of ATG8 interactors or sets of proteins already reported to be putative autophagy targets from proteomics or proteomics and transcriptomics studies. We have made new Figure 4 and 5 based on our data and these five other publications.

Point 8. Besides the technical challenges, the paper also tried to describe too many, sometimes unrelated observations. For example, the EM studies appeared unrelated to the protein turnover story and the phosphate response appeared disconnected from the attempt to demonstrate that the 14N/15N-labelling strategy provided a strong approach to understanding how autophagy controls protein abundances. A focused effort on a main story with appropriate controls and alternative strategies would improve the work immensely.

RESPONSE: We appreciate the suggestions. We have changed the way we show our major findings. We moved the original Fig 3, 4 and 6 to supplementals as they represent process rather than biological claims. We have made new Fig 4, 5 6 and Fig S9. We have tried to further focus on the findings revealed by combined analysis of protein abundance and degradation changes. We tested Pi starvation and found it has mild effect in protein turnover we observed. We think that while the effects are mild, this is important to outline, as nutrient starvation is often used to reveal autophagy mutant phenotypes and shapes discussions on the function of autophagy in plants.

Point 9. The metabolomic studies concentrated on amino acids, so it was hard to make any conclusions about other metabolites (organic acids) with the exception of glucosinolates. As a testament to this low coverage, lines 375-387 appear to be a collection of random thoughts on how autophagy might impact Arabidopsis metabolism.

RESPONSE: We appreciate the criticism of the reviewer, we don't want to be presenting random thoughts. We have rewritten the mentioned parts to focus on the effects of Pi limitation, to show that our Pi limitation is indeed a deficiency that plants are responding to.

<u>Reviewer #3:</u>

There are a number of cellular processes by which proteins turnover however large-scale identification of how specific proteins are turned over remains a challenge. Li et al. set out to identify proteins turned over through autophagy. Previous studies have looked identifying autophagic cargo by proteomics by looking for proteins that increase in abundance in autophagy mutants. However, as highlighted by the authors this is complicated by increases in ribosomal proteins in autophagy mutants. Here the authors use their established method that uses incorporation of stable isotope (N14 vs N15) to measure protein degradation. Ultimately, using quantitative proteomics to determine proteins with alerted stability in autophagy mutants they identify potential autophagic cargo proteins. They provide thorough descriptions of cellular differences as well as between root and shoot tissues and expand these analyses to Pi limiting conditions. Overall, this is a robust study that provides a wealth of quantitative information for other labs to purse but also reports insightful biological conclusions.

Point 1. The authors quantify ~1000 or so proteins in the each of their experiments. Perhaps a brief discussion of how measure only the most abundant proteins may or may not influence their conclusions would be useful.

RESPONSE: We agree with the reviewer suggestion. We have added a sentence into the result section to clarify this.

Point 2. How accurate is single point measurement for calculating degradation rate?

RESPONSE: Please see responses to Reviewer 2.

Point 3. Line 87: Translation is also a step in gene expression. Perhaps instead of "gene expression" use "transcription".

RESPONSE: We have corrected.

Point 4. Figure 1 legend needs edited. Last sentence reads "An explanation of values defining each group would be important."

RESPONSE: We have removed the sentence.

Point 5. Line 670: Seeds were stratified for 2-3 days rather than vernalized.

RESPONSE: We have corrected.

Point 6. Information on mass spectrometer used for proteomics and acquisition settings is lacking.

RESPONSE: We have added more of this information or refer to previous publications as appropriate.

TPC2021-RA-00373R1 2nd Editorial decision – *accept with minor revision* February 10, 2022

We have received reviews of your manuscript entitled "Defects in autophagy lead to selective in vivo changes in turnover of cytosolic and organelle proteins in Arabidopsis." Thank you for submitting your best work to The Plant Cell. The editorial board agrees that the work you describe is substantive, falls within the scope of the journal, and may become acceptable for publication, pending revision and potential re-review. I should note that we do not commonly request a second round of revision. However, given the general support of your study by the reviewers, we would like to give you the opportunity to address the remaining criticisms.
As you will see, two of the three reviewers are satisfied with the revisions, while reviewer #2 remains critical. In post-review consultation, these remaining criticisms were shared by all three reviewers, and therefore it would be premature to move ahead with the current version. The reviewers agree that several issues need to be clarified and perhaps elaborated upon:
1. The number of differential proteins in the tag mutant is considered very small, particularly when comparing to earlier datasets. This small number also makes it hard to draw conclusions about the role in nutrient starvation. The study would benefit from more extensive visualisation of the variability of protein quantities among replicates. This would help show the reproducibility of measurement.
2. It would be important to more explicitly contrast/compare the dataset to the earlier dataset on atg2. Even if methods are different, the number of proteins flagged as differential is vastly different.
3. We would not consider further validation (Western blots, qRT-PCR, GFP-ATG8 assay) critical, but anything that can be added along these lines will likely strengthen the conclusions.
Please note that a revised version will be sent back to the same reviewers. We would therefore encourage you to address the criticisms as fully as reasonably possible.

TPC2021-RA-00373R2 2nd Revision received May 9, 2022

Reviewer comments and **author responses**:

Reviewer #1:

My main concern was the lack of validation of the large datasets in the manuscript. The authors have validated some of them in protoplasts.

Point 1. Even though I have serious doubts over the value of protoplast validation, they have addressed my points, so I have no further suggestions.

RESPONSE: To extend validation further, we have performed western blotting in wild type, *atg5* and *atg11* lines to validate our findings that FBA8 accumulated in autophagy mutants (FigS12). A further *atg7* background control was added to the protoplast validation (Fig6). New and previous experimental evidence all support vacuolar degradation of FBA8 through autophagy. We thank the reviewer for their positive comments and constructive suggestions.

Reviewer #2:

As stated in a previous review, this manuscript attempts the laudable task of attempting to identify autophagy cargo through 14N/15N labelling of the total protein in WT and atg mutant backgrounds, and then measuring the isotope ratios of individual peptides by MS as an indication of the turnover for specific proteins. While we agree that such measurements would be helpful in defining autophagy targets, we are not convinced that the authors have accomplished this objective.

Point 1. While we are not in the position to determine if such end point assays are sufficient to determine widely varied turnover rates (especially given the length of the labelling was 3 days), even if the method worked, so few proteins (only 122 total as stated in line 335) were determine to be targets based the fold changes measured (atg mutant versus WT) that it raises the question whether the authors are seeing what they think they are seeing. For example, Figure 3 says that only 4 peroxisomal and 10 ER proteins were determined to be affected. Could it be that these proteins were actually degraded in the cytoplasm as they awaited transport into these organelles as opposed to degradation of the whole organelle. Or more likely could it be the stochastic nature of MS detection generated such differences. The authors really need to show scatter plots of their biological and technical replicates to confirm that measurement of such small changes is possible and statistically relevant by showing no changes when the biological replicates for the same treatment are compared. This same problem with low level detection also compromises the Pi studies. For example, in WT, the authors only found 6 proteins that were degraded faster under Pi limiting conditions versus well feed. This could easily be attributed to noise in MS detection and quantification. There is also not enough data to support the claims that low Pi leads to autophagic degradation of mitochondria and chloroplast by a process involving atg11. The box plots in Figure 9 look very similar and the PCA analyses are strangely overlapping. Again, no support from western blotting was included even though antibodies are available.

RESPONSE: We thank the reviewer for these questions. Some of these questions had been answered in our previous revision. For example, the reason we chose 3 days ^{15}N labelling for protein turnover rates measurements, protein turnover measurements procedures and the 'piece meal' versus 'engulf as a whole' questions have been answered before. Also we had performed a systematic correlation analysis for protein abundance and protein turnover datasets for separate biological replicates as requested in the previous revision (Matrix correlation analysis in Data S1, DataS3). Comparative statements about our protein abundance measures with those of other papers using different mass spectrometry platforms suffer from the problem of comparing unlike things and then noting differences. To provide a more direct comparison of what a traditional label free quantification (LFQ) analysis of our protein abundance would look like, we have performed such an analysis for direct comparison to our chosen analysis method. Label free MS1 gives higher coverage of proteins compared with ^{15}N spike-in method, as expected. We have added label free MS1 measurements to DataS1 and DataS7. However, we chose to use ^{15}N spike-in data because our pipeline uses stricter filters to remove low quality data (also see our response why we can confidently ignore the fractionation procedures in the 1st revision), but this also lowers the number of proteins we report in the manuscript. We have provided a few new supplementary figures to show the independent LFQ data and reproductivity of our protein abundance measurements (Fig S2, Fig S4 and Fig S14). We note that it is still a challenge to measure protein abundance using ^{15}N spike-in with the same type of coverage as label free/MS1 quantification because labelled sample produce more complicate mass spectrum than label free strategy. However, our main focus of this study was a combined analysis of protein abundance and turnover changes. ^{15}N spike-in produce similar coverage to ^{15}N progressive labelling turnover data and similar sets of peptides backing that comparison. We thus choose to report high confident protein abundance data and combined it with measured turnover rates in this study.

Point 2. While the Reviewers agree that comparisons of protein to mRNA levels do not tell a complete story, it would have been good for the authors to test some of their candidate targets seen in their 14N/15N approach (WT versus atg7) by qRT-PCR and compare the fold changes to those seen with the proteins by MS.

RESPONSE: We have not sought transcriptional validation of a post-translational process because we don't think this provides strong evidence of whether or not a protein is degraded at a different rate. Instead, we have used two independent MS-based strategies for protein abundance measurements coupled to evidence of change in the turnover rate. In our view this is superior point of comparison to address the direct issue of autophagic degradation. Looking for transcriptional responses in an autophagic mutant background can easily be secondary and independent phenomena that mask targets or mistake targets. Label free and ^{15}N spike-in separate protein

abundance measurements give very good correlations (Fig S5) and our point is not to focus on these alone but only when they can be related to changes in protein turnover rate. Previous reports from a range of researchers (included ourselves, Plant J 2021 108(2):459-477 doi: 10.1111/tpj.15452) have shown mRNA to protein comparisons show poor correlations in autophagy mutants, and it is very unclear that filtered sets from such comparisons are high fidelity tools to find autophagy targets. In the case of FBA8, we have identified it with our protein-based strategy and confirmed it with western blotting for FBA8 abundance in Col-0, *atg5* and *atg11* and protoplast studies with labelled FBA8.

Point 3. The Discussion is 8 pages long with way too much speculation. Such speculation can also be seen through the Results that names a litany of proteins suspected to autophagy targets without additional experimental support. See lines 543-572 and 616-630 for examples. These sections could be condensed significantly. Similarly, 5.5 pages were devoted to references, which together with the point above leads us to conclude that the paper is trying to say too much with not enough meaningful data. Other examples are the microcopy in Figure 2 and the GC-MS data in Figure 7. For the microscopic data, no statistical support was provided showing that the small changes see were relevant. At a minimum, the authors should also try to relate the metabolomic data to the proteomic data. Maybe it is only a specific organellar pathway or complex that is degraded by autophagy and not the entire compartment. A minor point is that some of the metabolite changes reported in the text were not observed in the heat map. Example: changes in fumarate, and increase in D2-hydroxglutarate.

RESPONSE: We thank the reviewer for the critics and suggestions. We agree with the reviewer that some of our observations from protein abundance and turnover deserve further exploration. While we had chosen glycolytic FBA8 as a case study as a validation of our data, there are still many interesting points that can be addressed by ourselves or other groups in future studies. We have tried our best to trim the discussion section. From our point of view, we need to provide readers with commentary to follow our hypotheses rather than just report a big or short list that get lost in supplementary material. As the reviewer commented, most readers are not mass spectrometry experts, so we seek to engage the reader with hypotheses and literature links to the observations we make. Regarding statistics and claims. We have performed ANOVA post-hoc Turkey test for the TEM images (Fig 2). Both fumarate and D-2-hdroxglutarate show significant increase under Pi starvation. The # mark means significant changes after Pi starvation. We had shown this in the figure legend (Fig7).

Point 4. It was good to see new data looking at a presumed autophagy substrate by another method, in this case by western blots. But the data for FBA8 are not convincing (1.4 to 1.3) when using relatively non-specific drugs to block autophagy (along with other processes). atg mutants would have been a better choice. Internalization of FBA8-DsRed into protoplasts was shown but many of the vacuolar puncta did not co-localize with the GFP-ATG8a marker and no mutant backgrounds were included even though plenty of atg mutants are available. Moreover, how is the partial co-localization explained?

RESPONSE: We appreciate these requests for validation. We have now used FBA8 antibodies for western blotting in *atg5* and *atg11* mutant lines and show a 1.7-2.7 fold increase in this protein (FigS12). This serves as an independent validation of protein abundance measurements. Furthermore, we have performed a protoplast based FBA8 degradation assay in an autophagy deficient *atg7* mutant line (Fig6). AZD8055 and ConA treatments can induce colocalized FBA8-DsRed with ATG8a-GFP autophagic bodies in vacuole in wild type but not in *atg7* mutant line. These new experiments all support our combined protein abundance and protein turnover discovery that FBA8 is an autophagy cargo.

Reviewer #3:

The authors have addressed all my comments. Very nice revisions all around!

TPC2021-RA-00373R2 3rd Editorial decision – *acceptance pending* May 30, 2022

We are pleased to inform you that your paper entitled "Defects in autophagy lead to selective in vivo changes in turnover of cytosolic and organelle proteins in Arabidopsis" has been accepted for publication in The Plant Cell, pending a final minor editorial review by journal staff. Please note that the original reviewer #2 was not available to comment on the revision, and we have asked the other two reviewers to evaluate your responses to reviewer #2's concerns and suggestions. As you will see, both are strongly supportive of moving ahead with the present version.

Final acceptance from Science Editor June 21, 2022
